# Nutritional Components in Western Diet Versus Mediterranean Diet at the Gut Microbiota–Immune System Interplay. Implications for Health and Disease

**DOI:** 10.3390/nu13020699

**Published:** 2021-02-22

**Authors:** Cielo García-Montero, Oscar Fraile-Martínez, Ana M. Gómez-Lahoz, Leonel Pekarek, Alejandro J. Castellanos, Fernando Noguerales-Fraguas, Santiago Coca, Luis G. Guijarro, Natalio García-Honduvilla, Angel Asúnsolo, Lara Sanchez-Trujillo, Guillermo Lahera, Julia Bujan, Jorge Monserrat, Melchor Álvarez-Mon, Miguel A. Álvarez-Mon, Miguel A. Ortega

**Affiliations:** 1 Department of Medicine and Medical Specialities, Faculty of Medicine and Health Sciences, University of Alcalá, 28801 Alcalá de Henares, Spain; cielo.gmontero@gmail.com (C.G.-M.); oscarfra.7@hotmail.com (O.F.-M.); alahoz1199@gmail.com (A.M.G.-L.); leonel.pekarek@gmail.com (L.P.); alejandrolivealcala@gmail.com (A.J.C.); natalio.garcia@uah.es (N.G.-H.); mjulia.bujan@uah.es (J.B.); jorge.monserrat@uah.es (J.M.); 2 Department of Surgery, Medical and Social Sciences, Faculty of Medicine and Health Sciences, University of Alcalá, 28801 Alcala de Henares, Spain; fernando.noguerales@uah.es (F.N.-F.); angel.asunsolo@uah.es (A.A.); 3 Department of General Surgery, Príncipe de Asturias Hospital, 28806 Alcalá de Henares, Spain; 4 Ramón y Cajal Institute of Sanitary Research (IRYCIS), 28034 Madrid, Spain; s.coca@uah.es (S.C.); larasancheztrujillo@gmail.com (L.S.-T.); 5 University Center for the Defense of Madrid (CUD-ACD), 28047 Madrid, Spain; 6 Unit of Biochemistry and Molecular Biology (CIBEREHD), Department of System Biology, University of Alcalá, 28801 Alcalá de Henares, Spain; luis.gonzalez@uah.es; 7 Service of Pediatric, Hospital Universitario Principe de Asturias, Alcalá de Henares,28806 Madrid, Spain; 8 Psychiatry Service, Center for Biomedical Research in the Mental Health Network, University Hospital Príncipe de Asturias, 28806 Alcalá de Henares, Spain; guillermo.lahera@gmail.com; 9 Immune System Diseases-Rheumatology, Oncology Service an Internal Medicine, University Hospital Príncipe de Asturias, (CIBEREHD), 28806 Alcalá de Henares, Spain; mademons@gmail.com; 10 Department of Psychiatry and Medical Psychology, Hospital Universitario Infanta Leonor, 28031 Madrid, Spain; 11 Cancer Registry and Pathology Department, Hospital Universitario Principe de Asturias, 28806 Alcalá de Henares, Spain; miguel.angel.ortega92@gmail.com

**Keywords:** gut microbiota, host immunometabolism, intestinal barrier, mediterranean diet, western diet, immunomodulation, food matrix, micronutrients, malnutrition

## Abstract

The most prevalent diseases of our time, non-communicable diseases (NCDs) (including obesity, type 2 diabetes, cardiovascular diseases and some types of cancer) are rising worldwide. All of them share the condition of an “inflammatory disorder”, with impaired immune functions frequently caused or accompanied by alterations in gut microbiota. These multifactorial maladies also have in common malnutrition related to physiopathology. In this context, diet is the greatest modulator of immune system–microbiota crosstalk, and much interest, and new challenges, are arising in the area of precision nutrition as a way towards treatment and prevention. It is a fact that the westernized diet (WD) is partly responsible for the increased prevalence of NCDs, negatively affecting both gut microbiota and the immune system. Conversely, other nutritional approaches, such as Mediterranean diet (MD), positively influence immune system and gut microbiota, and is proposed not only as a potential tool in the clinical management of different disease conditions, but also for prevention and health promotion globally. Thus, the purpose of this review is to determine the regulatory role of nutritional components of WD and MD in the gut microbiota and immune system interplay, in order to understand, and create awareness of, the influence of diet over both key components.

## 1. Introduction

The term microbiota refers to the set of microorganisms that inhabit superior organisms, including human beings, constituting a unique entity named a holobiont [1]. During the last few decades, interest has been growing in the knowledge of the microbial ecosystem, as it is a crucial component of the individual’s health, keeping a homeostatic balance with the host [2]. Recent advances in sequencing techniques are playing a critical role in the study of the relative abundance of microorganisms in the different environments of the human body, bacteria being the most common members of the microbiota (90%), although viruses, fungi, archaea and even protozoa have also been detected. Even so, the complete microbial structure is far from complete elucidation [3]. Not only are the populations of microorganisms important points of study, but so too are their genome and the end-products of their metabolism, all of these being collected under the term microbiome [4]. Microorganisms have been identified in various locations of the human body, such as the oral cavity, upper airways, genito-urinary tract and skin. However, it is in the gastrointestinal tract, and particularly in the gut, where they are most relevant, and are directly related to a broad spectrum of pathologies [5].

The important effects of gut microbiota in the organism are partly due to the tight relationship sustained with the host’s immune system from the early stage [6]. This interaction is bidirectional and dynamic, equally representing a key part of the knowledge of the different homeostatic and pathophysiological conditions [7]. Scientific evidence has supported the direct influence of diet in the gut microbiome and immune system, and a large number of studies are being developed to modulate both components, this balance being considered a central aspect of precision nutrition [8,9]. In addition, interventional nutrition is another study issue, offering to people with established pathologies such benefits as probiotics, prebiotics or bioactive compounds, providing a potential approach to influence the microbiota–immunity dialogue [10]. Thus, the purpose of this study is to review the impact of nutrition and diet on the gut microbiota–immune system communication, exploring the basis of their interplay and the consequences of diet in this relationship. 

## 2. The Human Gut Microbiota

### 2.1. Structure, Diversity and Dynamics of the Gut Microbiota

As previously reported, all domains of microorganisms compose the gut microbiota, including bacteria, archaea, viruses, fungi and protozoa. According to the current evidence, the ratio found between microorganisms and human cells is approximately 1:1 (3.8 × 10^13^ vs. 3 × 10^13^). This relation is noticeably higher when analyzing the genomes, microbial genetic content being 100 to 200 times superior to the content of our cells [11]. More accurately, recent studies have quantified the bacterial genes in the gut, finding up to 22 million genes in this location. Moreover, these genes were also determined to be different among individuals, being denominated as singletons, thus demonstrating the interpersonal phenotypic heterogeneity of the human gut microbiota [12]. Due to the representative number and the various vital functions of the gut microbiota, some works, such as the Human Microbiome Project conducted by the National Institute of Health (NIH) of the United States, have been developed to provide standardized data regarding the human microbiome in physiological conditions and diseases, recognizing a “microbial core”, which is fundamental to the health of the individual [13]. Bacteria are the major components of the microbial core. In fact, up to 2776 species of bacteria have been recognized in the human gut, including 11 different phyla [14].

Firmicutes are the most extended bacterial family (65%), followed by Bacteroidetes (23%) and Actinobacteria (5%) [15]. Among the Firmicutes group, it is worth noting the presence of type IV and XIVa Clostridia, belonging to the genus Clostridium, Ruminococcus and Eubacterium, whereas Bacteroides and Prevotella are the most important components of Bacteroidetes [16]. Frequently, the higher or lower presence of some of these microorganisms may lead to the classification of the individual’s microbiota into enterotypes, which are the following: enterotype 1, with augmented levels of Bacteroides and reduced Prevotella; enterotype 2, with decreased Bacteroides and increased Prevotella, and enterotype 3, enriched in Ruminococcus [17]. Importantly, this classification is non-exclusive, but it is useful to simplify the study of gut microbiota, keeping a tight connexion with diet or the individual’s physiology [18]. The Bifidobacterium genus is the principal member of the Actinobacteria phyllum [19]. Proteobacteria is the fourth-ranked phylum in terms of abundance, characterized by its Gram-negative staining with lipopolysaccharides (LPS) in the outer membrane. In this group, the most important examples are Escherichia and Helicobacter [20]. In the same manner, other members with less abundance hold the same importance in the gut microbiota, as is the case of *Akkermansia muciniphilla*, the only species of Verrucomicrobia [21]. 

On the other hand, viruses are additional components of healthy gut microbiota, shaping what is known as the virome. The virome is equally unique and stable, and is influenced by the bacterial composition [22]. In fact, approximately 45% of mammal viruses may compose the virome of healthy individuals, with no clinical outcomes, although in other situations these viruses may interact with other microorganisms, leading to an infectious disease [23,24]. However, bacteriophages are the most abundant viruses in the gut, particularly the crAssphages, which under homeostatic conditions are usually found as inactive prophages. Even so, when having a disease status, an increased activation of the prophages and their lytic activity has been observed [25]. Plant-viruses and giant viruses may also appear in the gut virome [26]. Likewise, Archaea, mainly represented by methane-producer microorganisms from the genus Methanobrevibacter, appear to play significative roles in the regulation of microbial populations in the human gut [27,28]. Fungi are another component of the intestinal microbiota, whose abundance is directly influenced by bacterial interactions [29]. Finally, more complex organisms, such as protozoans and even helminths, might provide substantial interactions with the gut microbiota and host homeostasis [30,31]. As bacteria are the major group of microorganisms residing in the intestine, this domain will be the subject of study when referring to the gut microbiota.

It is crucial to understand that the gut microbiota represents a dynamic structure in continuous communication with the environment. To favor their ecological niches, microorganisms serve themselves via symbiotic relationships with the host, including mutualism or commensalism. In the first case, both microorganism and host are benefited, whereas in commensalism only one part is favored without harming the other [32]. In this context, a pathobiont would be a microorganism that under physiological conditions has no negative effects, but in pathological situations may be detrimental to the individual’s health [33]. Likewise, microorganisms may interact among themselves, enhancing or limiting the growing of certain groups. A critical part of these intra and interspecies relationships is quorum sensing, which may determine the behavior and composition of the microbial populations [34,35]. Another means of communication is through the production of antimicrobial compounds, capable of inhibiting the growing of other microorganisms. It is worth noting the role of bacteriocins secreted by some species from the genera of Lactobacillus and Bifidobacterium, which negatively influence the establishment of pathogen bacteria such as *Listeria monocytogenes* or *Clostridium perfringens* [36,37]. In summary, the gut microbiota is a diverse, unique and dynamic element in the human body, and it will be essential for the physiological/pathological status of the individual. 

### 2.2. Eubiosis vs. Dysbiosis: The Two Edges of Microbiota in Health and Disease 

The term eubiosis is used to describe the favorable physiological status of gut microbiota, with so-called “good bacteria” that are capable of controlling “bad bacteria”. The opposite situation, dysbiosis, could be defined as a loss of this beneficial homeostatic balance [38,39]. 

Under eubiosis conditions, the gut microbiota perform a broad variety of functions within the holobiont, essential for its health. This is due to the production of critical compounds such as short chain fatty acids (SCFAs), acting as local and systemic signaling molecules, with important implications in health and disease conditions [40]. The main examples of SCFAs are acetate, propionate and butyrate, obtained from the digestion of resistant starches and dietary fiber. Acetate production is widely distributed among many bacterial populations, whereas propionate and butyrate are restricted to certain microorganisms belonging to the phylum Firmicutes or *Akkermansia muciniphilla* [41]. Moreover, other products, such as lactate, obtained from the fermentation of non-digestible compounds of dietary fiber, could equally influence the production of propionate and butyrate [42]. Intestinal bacteria also actively participate in the metabolism of aminoacids, such as tryptophan, a critical element with important implications for the metabolism of serotonin, melatonin or kynurenine, and having consequences in other physiological processes [43]. Likewise, gut microbiota are essential for the synthesis of vitamins, such as vitamin K or many of those of the B complex, the degradation of polyphenols from the diet, xenobiotic elimination, and even the metabolism of bile acids (BA) [44,45]. All these components interact bidirectionally with the gut microbiota. In other words, the gut microbiota produce and control these products, and the presence of the different elements modulates the microbiota composition [46]. Some authors refer to the “phylometabolic core” instead of the phylogenetic core, as it better reflects the metabolic functions performed by certain groups of microorganisms, including butyrate or propionate producers and lactate users, as well as bacteria involved in bile acid metabolism or vitamin synthesis, and much more [47]. 

In the same manner, in recent years, the central role of the microbiota in gut–brain communication has been established, shaping the microbiota–gut–brain axis, as many of these metabolic products serve as a method of dialogue between both organs, indirectly through intermediate mediators, or directly by the vagus nerve and the enteric nervous system [48]. This relationship also occurs with other structures in the body, such as bones [49] or the cardiovascular system [50], thus showing the prominent effects of the gut microbiota and their metabolites on the individual’s health. Thus, the gut microbiota may favorably interact with the host cells, leading to a healthy homeostatic status, or may act negatively, contributing to an inflammatory response under dysbiosis conditions. On the other hand, gut dysbiosis may play an essential role in the development of a broad spectrum of pathologies, such as neurodegenerative diseases, metabolic disorders, inflammatory and autoimmune diseases, among others [51]. Nevertheless, whether these changes are the cause or consequence of different effects remains unclear [11,52], although recent research seems to indicate that both are correct, as gut dysbiosis contributes both to a pathophysiological mechanism and the coadaptation to unfavorable conditions [53]. Accordingly, two types of dysbiosis can be distinguished: taxonomic and metabolic dysbiosis.

Taxonomic dysbiosis is manifested as a quantitative or qualitative loss of the gut microbiota’s composition. This is closely associated with a reduced microbial diversity, frequently presented as an altered Firmicutes to Bacteroidetes ratio [54]. The altered proportion of both bacterial phyla has been reported in different pathologies, including infections [55] and non-communicable diseases (NCDs), such as type 2 diabetes mellitus (T2DM) and obesity, in which a simultaneous decrease in bacteria belonging to the phylum Bacteroidetes, and an increase in non-favorable Firmicutes bacteria from the genus Clostridium, has been reported [56,57]. Although many works have focused on the study of the Firmicutes to Bacteroidetes ratio, recent research shows that this imbalance is not enough to obtain a complete assessment of the gut microbial environment [58]. The complementary study of the remaining communities, such as Proteobacteria [59], Actinobacteria [60], or the less abundant but important bacteria *Akkermansia muciniphilla* [61], is also important in the taxonomic study of the gut microbiota.

Similarly, metabolic dysbiosis is used to express not a change in the taxonomic composition, but an altered phylometabolic core [47,62]. To characterize the type of dysbiosis in each individual, different methods can be used, including techniques of metagenomic sequencing to unravel the microbiome composition [63], or metabolomic approaches such as mass spectrometry, which can be used to examine the metabolic profile, for example through fecal samples [64]. Independently from the type of dysbiosis, important alterations in the synthesis of microbial metabolites have been reported due to this altered microbiota, with negative repercussions for the host [54]. These effects of gut microbiota are mediated or influenced by the immune system, which responds in the same manner as the gut microbiota [65]. One of the most worrying effects of gut dysbiosis is the presence of a component from the outer membrane of Gram-negative bacteria in the bloodstream, known as endotoxin or Lipopolysaccharide (LPS), which is associated with chronic inflammation [66].

As such, it is crucial to describe the different cellular and structural elements in the gut ecosystem in order to understand how the gut microbiota–immune system relationship works.

## 3. Integrators of the Gut Mucosa and Immunobiology of the Gut

The gut, just like the rest of the digestive structures, is composed of different layers, including the epithelium, lamina propria, muscularis mucosa (these three layers forming a combined layer known as the mucosa), submucosa, muscularis propria, and an adventitia. Structurally, two main divisions could be distinguished: the intestinal villi, implicated in the absorption and transport of nutrients and crypts, where stem cells are located [67,68]. The large intestine does not present intestinal villi. The gut epithelium is mainly formed by the epithelial cells known as enterocytes, cells specialized in the absorption of nutrients and the entry of substances from the intestinal lumen into blood, and which thus assume a key role in the intestinal barrier and antigen uptake [69]. Globet cells are equally important elements of the gut epithelium, particularly in the large intestine, and are responsible for the production and secretion of mucine, a glycoprotein acting as a protective agent that prevents the entry and invasion of microorganisms in the different gut layers [70]. Similarly, other components of the epithelium need to be mentioned here, including tuft cells, specialized in the immune responses against eukaryotic parasites and acting as a gut microbiota sensor for the host [71,72], or the Paneth cells, specifically located in the intestinal crypts, responsible for the production of antimicrobials peptides, therefore controlling gut microbiota composition [73]. Although they are normally restricted to small intestine crypts, and at very low proportions in the first regions of the large intestine (from cecum to transverse colon), Paneth cells can also be found under inflammatory pathological conditions, such as inflammatory bowel disease (IBD), in the final portions of the large intestine, termed as metaplastic Paneth cells [74]. Enteroendocrine cells (L-cells) are hormone-producer cells, regulating the appetite, gut microbiota composition and the integrity of the intestinal epithelium [75].

Immediately under the epithelium, the lamina propria is the next layer belonging to the mucosa. Here, a plethora of immune cells are found and organized in lymphoid tissues known as GALTs (gut-associated lymphoid tissues) [76]. It is an extraordinarily complex system, that has coevolved and developed with a wide variety of microorganisms, maintaining a continuous dialogue with them, equally assuming important functions in cellular nutrition, antigen tolerance, and energy starvation in the host [77]. GALT is a type of MALT (mucosa-associated lymphoid tissue), with essential functions in gut homeostasis. The small and large intestinal surface represents a 32 m^2^ structure, with an estimated 10^14^ commensal microbes and more than 30 kg of food proteins measured yearly, which may be controlled and tolerated by GALT [78,79]. The number of cells in the GALT increases progressively from the duodenum to the final portions of large intestine, according to the higher presence of microorganisms. Different immune cells can be detected forming the GALT, including T and B lymphocytes, dendritic cells (DCs), and macrophages, among others. These cells are dynamically recruited from the body to the gut, depending on different stimuli under conditions of health and disease [80]. In addition, there are intraepithelial lymphocytes located at the intestinal epithelium, interacting with the different microorganisms and cells in the gut [81]. There are different denominations of GALT depending on the location, including Peyer’s, cecal, or colonic patches. In communication with GALT, in the follicle-associated epithelium overlying the surfaces of intestinal lymphoid tissues resides an additional cellular type named microfold (M) cells [82]. M cells play a key role in the capture and translocation of microbes and molecules from the intestinal lumen, subsequently recognized by DCs, then interacting with T and B cells, leading to a regulation of the inflammatory response and secretion of immunoglobulin A (IgA), thereby modulating microbial communities [83,84]. 

**Figure 1:**
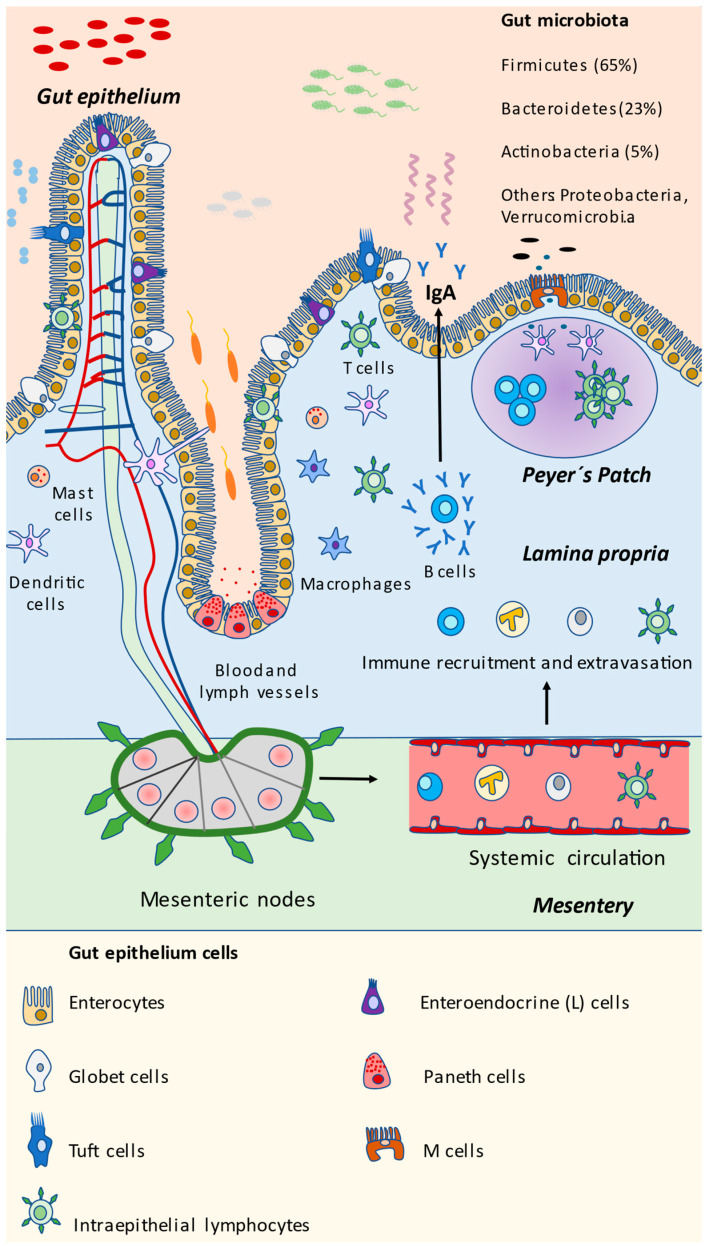
Microbial and eukaryotic components of the gut. As represented in the scheme, gut mucosa is a highly dynamic structure in which microorganisms and epithelial and immune cells are interacting continuously. The gut microbiota is mainly composed of the Firmicutes phylum, followed by Bacteroidetes, Actinobacteria, and other bacteria that are less abundant but equally important, including Proteobacteria and the Verrucomicrobia *Akkermansia muciniphilla*. Immune detection and response may be assessed at the local Peyer’s patch present in the small intestine, or through mesenteric nodes, activating immune cells and accessing the gut through the bloodstream, finally promoting the release of IgA by plasmatic B cells. Other cells may aid in the capture of microbial antigens, coordinating the immune response, including tuft cells, M cells and even intraepithelial lymphocytes. Paneth cells prominently act through the release of antimicrobial compounds. Enterocytes and globet cells play a key role in maintaining the intestinal barrier, whereas enteroendocrine cells produce certain products that are essential for metabolism and the individual’s health.

The different bacterial and eukaryotic cells in the gut are summarized in Figure 1. Now that the components of the intestinal mucosa have been identified and described, we will further explore the different mechanisms by which they interact with gut microbiota, representing a potential approach to modulating and disentangling potential mechanisms for controlling the gut microbiota and its interactions with the immune system in the gut.

## 4. Basis of Gut Microbiota–Immune System Interplay

### 4.1. Microbial Communities and Their Products

The different cells located in the epithelium of the intestinal mucosa, along with the immune cells, contain various receptors for different molecules, known as pattern recognition receptors (PRRs), specialized in the detection of MAMPs (microbe-associated molecular patterns), and also named PAMPs or pathogen-associated molecular patterns [85,86]. Among the most important PRRs are membrane toll-like receptors (TLR) or C-lectin receptors (CLR), and the cytosolic NOD-like receptors (NLR) [87]. Similarly, metabolic products from the microbial communities will be captured and recognized by these cells, with important implications for gut homeostasis and immunity [88]. Thus, both the gut microbiota and immune systems affect each other through bidirectional interactions. This relationship provides potential benefits to the host, for example in the education and regulation of immune functions and the formation of the intestinal barrier [89,90]. On the other hand, there is evidence supporting the interpretation that the interaction of the intestinal microbiota and the immune system cells may damage the intestinal barrier, increasing bacterial translocation with systemic pro-inflammatory effects [91]. This pathogenic mechanism has been observed in different diseases [92,93,94]. Here, we will summarize some of the mechanisms and signaling processes used by bacteria and the possible responses enacted by immune cells. 

Some microbial products have been established to act as immune activators. For instance, LPS and its lipid A domain abundantly expressed by Gram-negative bacteria are recognized by the innate immune system, leading to a pro-inflammatory response [95]. Likewise, some types of bacteria including certain members of Bacteroidales, Erysipelotrichales, Clostridiales, and Bacillales are capable of producing capsular polysaccharides, which may encompass similar mechanisms. This is the case of polysaccharide A (PSA) located in the capsule of *Bacteroides fragilis*, which may be recognized by plasmacytoid DCs promoting an anti-inflammatory response in the gut [96]. The recognition of these components contributes to the release of IL-10, the most important anti-inflammatory cytokine mainly produced in the gut by T regulator lymphocytes (Treg) [97,98]. Other bacteria, such as segmented filamentous bacteria (SFBs), may have distinct effects on the immune response. SFBs are a group of bacteria bound to the intestinal epithelium in a mutualist relationship, up-taking available nutrients while releasing vital antigens with a strong immunomodulatory effect on the host [99]. Thus, SFBs are related to an immunocompetence status, and are also associated with the production of IgA by B cells in the gut [100] and even in extraintestinal locations, collaborating in the proper response against fungal pathogens in the lungs [101]. These effects are coordinated by Th17 cell polarization, which is key to maintaining host homeostasis [102,103]

The balance between different T cells populations appears to play a central role in the physiological and pathological conditions. For example, the balance between Treg lymphocytes (anti-inflammatory) and Th17 (pro-inflammatory) cells is vital for a proper inflammatory response, and it is prominently modulated by the gut microbiota [104]. Thus, a disruption in this balance, with reduced Treg and increased Th17, may be associated with intestinal pathology [105] or autoimmunity [106]. Similar to SFBs, there are other bacteria that may present immunomodulatory properties via adhesion to the intestinal epithelium cells, such as *Bifidobacterium adolescentiis*, *Escherichia coli* O157 or *Citrobacter rodentium*, which equally induce Th17 differentiation [107,108]. 

Other critical microbial populations include the bacteria from the genus Alcaligenes. Alcaligenes are opportunistic bacteria residing in the GALTs, similar to those in the Peyer’s patch, where DCs recognize their LPS through TLR-4 activation to foster the activity of IL-6 and IgA production [109]. Regardless of the fact that the systemic presence of LPS in the bloodstream (endotoxemia) is related with chronic inflammation [110], the presence of Alcaligenes in the GALT means that LPS interacts symbiotically with DCs in healthy individuals [111]. In addition, other bacteria, such as Clostridia, are a class of bacteria belonging to the phylum Firmicutes, and clusters IV and XIVa represent an important part of the healthy gut microbiota (10-40%), with important implications for the host [112]. These clusters, along with the XVIII, play a prominent role in the induction of Treg cells in the gut, thus promoting anti-inflammatory effects [113]. Nevertheless, there are other members of Clostridia, such as *Clostridium difficile*, that are not favorable for the immune system, the most common being those associated with dysbiosis [114]. 

Likewise, the activation of NLRs plays a prominent role in the regulation of microbial signatures. For instance, some members of this family, NOD-, LRR (leucine-rich repeat)- and pyrin domain-containing 6 (NLRP6) and NLRP3, are able to form multiprotein signaling complexes known as inflammasomes [115]. Multiple cells in the gut present inflammasomes, the activation of which is regulated by gut microbiota, thereby promoting the release of IL-1β and IL-18, pro-inflammatory cytokines responsible for antiviral responses in the gut or mucus secretion by globet cells, among other things [116]. It seems that the activation of the inflammasome is an essential form of communication between the host’s immunity and the pathobionts in the gut microbiota, such as *Proteus mirabilis* or *Helicobacter pylori* [117,118]. On the other hand, a deficiency in the inflammasome also promotes the overgrowth of certain microbial communities, meaning it is associated with intestinal inflammation [119,120]. 

The other bacteria playing a critical role in host homeostasis include *Akkermansia muciniphilla,* a special member of the gut microbiota involved in innate immunity [121] and in the adaptative immune response [122], increasing the levels of IL-10, and modulating T cells fate and IgG1 levels depending on individual characteristics, the composition of the gut microbiota and the interaction with additional environmental factors. This behavior is similar to those seen in beneficial bacterial strains, such as *Faecalibacterium prausnitzii* A2-165 and *Lactobacillus plantarum* WCFS1 [123,124].

Finally, some probiotic bacteria, such as *Lactobacillus rhamnosus GG*, *Lactobacillus casei Shirota*, *Bifidobacterium animalis Bb-12*, *Lactobacillus johnsonii La1*, *Bifidobacterium lactis DR10*, and *Saccharomyces cerevisiae boulardii*, are powerful inductors of the immune response, activating a wide variety of immune cells in a strain-specific and dose-dependent manner [125]. Due to their immunomodulatory properties, probiotics have been proposed as a potential adjuvant in certain pathologies, including cancer [126] or colitis [127], and even in healthy aging [128], supporting the use of these microorganisms for controlling the immune system. As a result, gut microbiota bacteria are thenceforth recognized by immune cells, leading to a proper response and the maintenance of gut homeostasis.

### 4.2. Microbial Metabolites

On the other hand, some microbial metabolites are components of note in the microbiota–immune system dialogue. SCFAs are components with powerful immunomodulatory properties. In the gut, enterocytes present different receptors to permit the entry of SCFA, which will be used in part to obtain energy by these cells [129]. Furthermore, SCFAs induce the release of TGF-β and IL-18 in the enterocytes, which are key activators of the inflammasome NLRP3 [130,131]. Moreover, there are also SCFA receptors expressed in the basolateral membrane, favoring the passage of SCFA to be distributed locally and systemically. In the gut, SCFAs may be recognized by G protein-coupled receptors (GPCR) such as FFAR-2, FFAR-3, GPR-41, GPR109A or Olfr78 [132]. This interaction will promote downstream cellular signaling, thereby regulating different products, such as hypoxia-inducible factor (HIF), collaborating with intestinal integrity and the production of antimicrobial substances by the Paneth cells [133]. In the same manner, SCFAs regulate the secretion of mucine by globet cells, or the release of GLP-1 (Glucagon-Like Peptide 1) and the peptide YY by L-cells, with important consequences for intake and insulin production [134]. In the immune cells, SCFAs have important effects on both innate and adaptative cells, leading to increased levels of IL-10, Treg and Breg cells [135,136]. Other studies have reported a boosted Th1 polarization in a non-pathological context, equally reducing Th17 levels [137]. On the other hand, gut dysbiosis may promote an increased Th1/Th17 balance, leading to IBD [138]. SCFA are also associated with the local production of IgA by B cells and with IgG systemically [139]. Besides this, SCFAs promote the activation of the histone-acetyl transferase (HAT) and the inhibition of the histone deacetylase enzyme (HDAC). Thus, SCFAs act as an epigenetic mechanism in the host, promoting an anti-inflammatory phenotype in the gut through the inhibition of the nuclear factor kappaB (NF-kB) [140]. On the other hand, the role of SCFA remains to be systemically elucidated. Recent works have shown that SCFA could activate the MAPK pathway, activating NF-kB and therefore presenting the opposite effects, favoring the overproduction of pro-inflammatory cytokines [141]. Importantly, this action is typically associated with pro-inflammatory molecules such as LPS or TNF-α. Thus, the global beneficial or detrimental effect of SCFA will depend on different factors, such as the eubiotic/dysbiotic status. 

Another key molecule in the gut microbiota–immune system dialogue is BA. BAs are produced by the hepatocytes and stored in the gallbladder. Then they are released in the gut, facilitating the emulsion, absorption, and digestion of fats. BAs are molecules derived from cholesterol, forming two main types of primary BA: cholic acid (CA) and chenodeoxycholic acid (CDCA) [142]. Then, primary BAs will be biotransformed by the gut microbiota, interacting with the farnesoid X receptor (FXR) or the G-protein coupled bile acid receptor 1 (GPBAR-1/TGR-5) [46], although it has been observed that secondary BA present a higher affinity with GPBAR-1 [143]. The different cells in the gut present both receptors in their membrane, having important immunomodulatory activity. For instance, BAs promote an anti-inflammatory M2 macrophage phenotype and reduce the M1 pro-inflammatory phenotype either in the gut or liver, with an increase in IL-10 levels and decreased IFNγ and IL-6 [144]. Simultaneously, secondary Bas such as taurine are powerful modulators of the NLRP3/NLRP6 inflammasome [145,146]. Other studies have shown the role of secondary BAs in the adaptative immune system, mainly through the regulation of the Treg/Th17 ratio [147,148] and Th1 populations [149]. BAs, in turn, act as important modulators of gut microbiota, acting in fact as antimicrobial compounds [150]. Alterations in the production of BA have been associated with dysbiosis conditions, leading to a loss of intestinal integrity, augmented bacterial translocation and the pathogenesis of inflammatory diseases [151,152,153]

Finally, tryptophan derivatives play a prominent role in the immunomodulatory capacity of gut microbiota. The tryptophan metabolism could be divided into three distinguished paths: (1) the kynurenine pathway, (2) serotonin synthesis and (3) the formation of tryptamine and indolic compounds [154]. Indole-derivatives and tryptamine have been proposed, and are considered critical products in the homeostasis of epithelial and immune cells in the gut, acting through the Aryl hydrocarbon receptor (AhR) [155]. The activity of indolic compounds synthesized by gut microbiota such as *Lactobacillus* sp. could stimulate the production of IL-22, thereby assisting the immune response against fungi such as *Candida albicans* [156]. In the same manner, indolic metabolites may promote the reprogramming of Th17 to Treg cells and other populations of adaptative and innate cells [157,158,159]. Ethanol-induced dysbiosis is associated with lower levels of indole-3-acetic acid (IAA), and indolic derivatives, leading to a reduced IL-22, hence promoting bacterial translocation and liver disease [160]. Importantly, some microbial species could also be involved in tryptophan synthesis, as well as participating in the formation of serotonin through this metabolite and in the regulation of systemic levels of tryptophan and the kynurenine pathway [161]. This could have important implications systemically, in different regions, including the brain or cardiovascular system [162,163].

**Figure 2:**
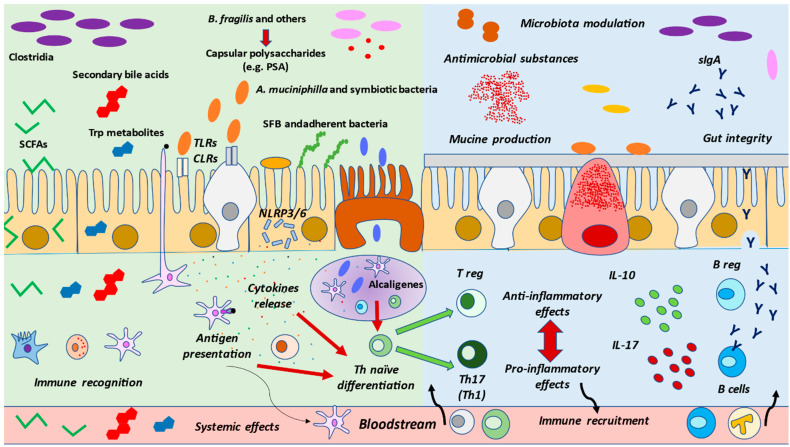
Interactions between gut microbiota and immune system. The presence of healthy gut microbiota, their products and metabolites are detected by the different cells located in the gut mucosa. DCs may up-take antigens and present them, as previously commented on, at the Peyer’s patch or mesenteric node, leading to Th naïve differentiation. Certain bacteria such as Alcaligenes may be found in the Peyer’s patch that equally regulate Th fate. Finally, some other bacteria may adhere to the epithelium, promoting the release of cytokines to modulate the immune response. These recognitions are mainly due to Toll-like receptors or Nod-like receptors, leading to inflammasome NLRP3/NLRP6 activation. Then, in gut eubiosis, the activated cells will conduct a proper response, which includes increased mucine production by globet cells, an augmentation of tight junctions by enterocytes, and the secretion of antimicrobial substances by Paneth cells or of IgA by B cells. Likewise, the balance between Treg/Th17 and pro-inflammatory and anti-inflammatory cytokines is vital for the regulation of immune responses, collaborating with an adequate, non-exacerbated response, but also with tolerance. Importantly, under pathological conditions, this balance is lost, and an inflammatory environment is created, contributing to the normal functioning of these cells, along with the associated gut dysbiosis.

Overall, the presence of certain types of microorganisms and their metabolites seems to be critical for host homeostasis and immunity, as summarized in Figure 2. Diet is a central regulator of both gut microbiota and immune cells. Thus, unraveling the connexion between nutrition, gut microbiota and immune system is essential in order to understand the link between the gut microbiota and the immune system 

## 5. Diet as the Main Modulator between Gut Microbiota and Immune System: Implications in Health and Disease

Diet is being described in the scientific literature as the most characterized factor that shapes gut microbiota and immune system, but other lifestyle factors should not be omitted, as well as exercise [164] and circadian clocks [165]. Diet has a rapid effect on gut microbiota composition, which promotes the growth of certain bacterial groups over others, as well as changes in intestinal pH, intestinal permeability, bacterial metabolites, and thus inflammation [63,65]. Macronutrients, in particular carbohydrates, seem to be the best described, whereas protein and fat hits are less well defined [166]. However, micronutrients are not a minor point to contemplate, given that vitamins deficiencies, for instance, alter barrier function and immune response in the GALT [167].

Immune system and microbiome interactions go together with diet. Human evolution has played a central role in these adaptations. However, it is important to understand that these changes should not be ascribed to certain foods or macronutrients, but to the entire diet composition, along with a higher consumption of plant-based sources [168,169]. Thus, malnutrition in occidental countries contributes to a state of chronic inflammation and metabolic problems, whereas the typical undernourishment of underdeveloped countries leads to nutritional deficits, and therefore immunodeficiencies [170]. 

For that reason, nutritional intervention has been proposed as a potential therapeutic approach, targeting both gut microbiota and the immune system [171]. As diet might be an easily malleable factor, diverse studies discuss the relevance of immuno-nutrition, which consists of modifying nutrient supply to modulate immune responses [172]. This is due to data collected from both nutritional intervention and observational studies, which show that nutrients alter immune biomarkers [173]. These days, these kinds of study are becoming more prevalent on account of the COVID-19 pandemic situation, which also proves that malnutrition contributes to a greater difficulty in recovering from infections [174,175], as well as to barrier function [176], where the microbiota status plays a key role in the immune response, even in these respiratory infections [177]. In general, diet induces changes, which could be referred to as nutritional programming, in the gut microbiota besides the metabolic and immune function [178]. In this context, the pathologies related to metabolism strongly depend on food intake, which is subject to the gut–brain axis that modulates appetite control [179], as well as to the microbial metabolites that interact with the satiety pathway, and in particular with hypothalamic neurons [180].

In addition, not only may nutritional modulation serve as an adjuvant treatment for diseases, but it also may contribute to prevention, or simply guarantee a better quality of life in healthy populations [8]. Animal models and 16S rRNA sequencing have allowed us to observe microbial diversity. Nevertheless, these studies have also demonstrated that changes in bacterial communities are much greater in the small intestine than feces when feeding animals with different diets [181], something particularly difficult to handle in humans.

In summary, dietary components can have a direct effect on the barrier added to gut microbiota populations and host metabolites, therefore modulating the immune system in the host [182]. However, it is important to understand that rather than the quantity, the quality of food and its nutrients is assumed to be the most determinant factor in a healthy/unhealthy diet. For instance, occidental diets are rich in refined and unhealthy products, and are poor in micronutrients such as vitamin A or D, and fiber, provoking the weakness of tight junctions and long-term inflammatory responses [183]. In contrast, the benefits of some functional components found in healthy diets, such as probiotics, prebiotics and polyphenols, are pursued in the restoration of gut health in disease [184]. All these components’ effects will be described with more detail below, in the context of a healthy model of dietary pattern (Mediterranean diet (MD)) versus an inadequate one (Western diet (WD)), in order to fully understand the role of different nutritional components in the gut microbiota and immune system’s interplay.

## 6. Mediterranean Diet as a Model of Healthy Eating 

MD every time shows more scientific evidence about its effects and benefits. This diet is characterized by a combination of highly complex carbohydrates in fiber (found in cereals, legumes, vegetables, fruits), polyunsaturated fatty acids with antiatherogenic and anti-inflammatory properties (found in olive oil and nuts), and bioactive compounds with antioxidative properties such as flavonoids, phytosterols, terpenes and polyphenols [185]. Similarly, a perfect balance of micronutrients, which are abundant in this diet, including vitamins and minerals, help avoid malnutrition and immunodeficiencies [186,187]. The immune system needs to cooperate with a group of substances to correctly perform its functions, and some of them are required in higher concentrations depending on the health status and the patient [188].

Nutrient-rich foods allow the body to repair inflammation triggered by nutrient-poor and high-calorie diets, contributing to attenuating cardiovascular risk factors [189]. Moreover, adherence to MD correlates with microbiota eubiosis reestablishment as Bacteroidetes and certain beneficial Clostridium groups grow, whereas Proteobacteria and Bacillaceae phyla decrease [183]. Thus, as the gut microbiota represents an indicative factor as regards the individual’s health status, it is also a factor that denotes adherence to a healthy type of diet, such as MD [190].

In certain trials from the PREDIMED (PREvención con DIeta MEDiterránea) study, gut microbiota status in feces was evaluated in the context of adherence to MD and nutrients consumption through 16S rRNA sequencing and quantitative PCR (qPCR) for metagenomics, and through high-performance liquid chromatography (HPLC) for metabolomics (mainly SCFAs analysis). The results showed that participants who consumed a greater amount of animal protein presented a higher Firmicutes:Bacteroidetes ratio and worse adherence to MD, whereas those who consumed less animal protein had a higher concentration of Bacteroidetes and a lower ratio, as well as better adherence to diet. Participants that consumed more polysaccharides and plant proteins showed higher concentrations of SCFA and better adherence [191].

WDs promote a pathological microbiota status, leading to an increase in Firmicutes:Bacteroidetes ratio, which can be attenuated by MD as the favorable bacteria and their metabolites’ production rises, whereas dysbiosis and LPS levels decrease. This fact is important in the management of certain pathologies from the dietary point of view, making MDa strategy to modulate host microbiota, causing different local and systemic responses [57]. This type of dietary pattern also associates with greater diversity, and better gut barrier function and permeability, than is seen for occidental patterns [192].

In fact, several nutritional intervention trials based on MD have collected the most relevant adaptations that this diet induces, including the following: reductions in lipid levels in serum; protection against oxidative stress; reductions in inflammation; platelet aggregation; modulation of hormones and growth factors implicated in cancer pathogenesis; and modulation of microbial metabolism, promoting the proper functioning of the host metabolism as well [193]. Nowadays, further work is being performed in order to prevent cancer, cardiovascular disease (CVD), and metabolic, or even infectious, diseases. Here, we will summarize the effects of the most relevant components of MD on the gut microbiota and immune system modulation. 

### 6.1. Monounsaturated and Polyunsaturated Fatty Acids

As a common characteristic of MDs, it is worth noting the abundance of mono-unsaturated fatty acids (MUFAs), and polyunsaturated fatty acids (PUFAs), with a very low consumption of saturated fatty acids, which has important consequences for both gut microbiota and immune system [194]. The largest known MUFA is oleic acid, which is found in the central component of MD extra virgin olive oil (EVOO). MUFAs are the main component of EVOO, representing an estimated proportion of 70 to 85% of the total content, and oleic acid occupying around 63 to 80%. Similarly, it also effects other MUFAs, such as palmitoleic acid, saturated fatty acids such as palmitic acid, or PUFAs, including linoleic acid (omega 6) and linolenic (omega 3), to quite a reduced extent [195]. Additionally, EVOO is enriched in other elements that will be subsequently discussed, such as polyphenols, carotenoids and tocopherols, fostering the expansion of beneficial bacteria [196,197]. Among these benefits, the high consumption of EVOO is known to boost lactic acid bacteria (mainly *Bifidobacterium* and *Lactobacillus*) and their bioactive metabolites in GALT, leading to a decrease in IL-6, IL-17A, TNF-α, IL-1β, COX-2, LDL-c, oxidized LDL-c (ox-LDL) and blood pressure [197,198], as well as modulating microbiota metabolism, encouraging butyrate production, possessing anti-inflammatory and atheroprotective properties [199], and protecting colonocytes against oxidative stress [200]. In the same manner, the positive effects of oleic acid on liver dysfunction and gut inflammation have been demonstrated [201]. Accordingly, MUFA should be recommended for healthy gut microbiota and immune system. However, despite its favorable effects, an excessive amount of MUFA may negatively alter the gut microbiota, decreasing the total number of bacteria [202]. 

PUFAs are another key element of MD, prominently represented by omega 3 and omega 6 fatty acids. Both are considered essential fatty acids and master regulators of the inflammatory response [203]. Importantly, PUFAs are key elements of the human membranes, and dietary intake of these fatty acids may have important consequences for health and disease [204]. Omega 3 fatty acids are extensively found in fish and seafood, nuts and seeds, plants oils, and fortified foods such as eggs or dairy products [205]. Omega 3 fatty acids include alpha linolenic acid (ALA; 18:3 ω-3), and their derivates, stearidonic acid (SDA; 18:4 ω-3), eicosapentaenoic acid (EPA; 20:5 ω-3), docosapentaenoic acid (DPA; 22:5 ω-3), and docosahexaenoic acid (DHA; 22:6 ω-3) [206]. On the other hand, omega 6 is highly concentrated in vegetable oils, nuts, seed, and soy derivates such as tofu, eggs or poultry. Linoleic acid (LA; 18:2 ω6) and arachidonic acid (ARA; 20:4 ω-6), which may be synthesized by proper LA or found in food sources [207]. Importantly, ARA is considered a precursor of pro-inflammatory molecules, including eicosanoid hormones, prostaglandins or leukotrienes, although the role of this omega 6 acid as a pro-inflammatory or anti-inflammatory component is controversial [208]. It seems that the global effect depends directly on the interaction with omega 3 fatty acids. It is assumed that a low omega 6:omega 3 ratio (no greater than 4:1) is related with anti-inflammatory effects in the immune system [209]. MD positively influences omega 6:omega 3 ratio, within a ratio of 2:1 to 1:1 maximizing the benefits of these essential fatty acids [210]. 

In this context, it is known that the gut microbiota are positively favored by the high presence of omega 3 PUFAs, balancing the Firmicutes:Bacteroidetes ratio and increasing favorable bacteria from the Lachnospiraceae and Bifidobacteria families, while limiting the growing of LPS-producing Enterobacteria, and thus having positive effects on the anti-inflammatory properties [211]. Moreover, it seems that gut bacteria are able to metabolize PUFAs, forming a broad spectrum of products that some authors considered microbial metabolites [212], such as CLA (conjugated linoleic acids), CLnA (conjugated linolenic acids), and non-conjugated fatty acids, such as vaccenic acid (*trans*-11-18:1). In fact, the capacity of the gut microbiota to inhibit the metabolized PUFAs from the diet is associated with a decreased risk of obesity and inflammation [213]. *Roseburia* spp. is an important member of the microbiota-metabolizing omega 6 fatty acids, and helps to to obtain CLA. Similarly, CLA formation occurs naturally in ruminants, in a process known as biohydrogenation, which permits the presence of this metabolite in meats and dairy products [214]. Then, CLA is recognized by the immune cells, enhancing the function of Treg [215]. In the same vein, *Lactobacillus plantarum* was found to produce CLnA, and to have important effects on gut microbiota composition by increasing Ruminococcus and Prevotella, leading to a reduced level of pro-inflammatory cytokines (TNF-α, IL-1β, and IL-6), and augmented expressions of anti-inflammatory IL-10 and the nuclear receptor peroxisome proliferator-activated receptor- γ (PPAR-γ) [216]

### 6.2. Fruits and Vegetables Rich in Polyphenols

Polyphenols are a group of secondary plant metabolites, divided into two subgroups, flavonoids and non-flavonoids [217], presenting antioxidant and anti-inflammatory properties, with effects on gut microbiota as well [218]. Soy isoflavones, cocoa flavanols, blackberry and raspberry anthocyanins, tea or nuts tannins, and a long list of polyphenols, boost the growth of beneficial symbionts such as *Lactobacillus* sp. [219], and inhibit opportunistic pathogens such as *Enterococcus caccae* [220]. Data collected from preclinical and clinical studies suggest not only an interference in quorum-sensing, but also the prebiotic effects of these phytochemicals in relation to beneficial bacteria, encouraging them to produce antimicrobials against pathogenic bacteria [221]. The tight junction dynamics in the epithelial barrier may be altered by interactions with different phenolic compounds: where some of them promote the expression of tight junctions, others reduce it, and both events contribute to the integrity of the semipermeable character [222]. In addition, polyphenols have been recently identified as a key modulator of tryptophan metabolism by the gut microbiota, which may aid in the clinical management of certain pathological conditions [223]

One of the main characteristics of MD is the abundance of fruits and the availability of aromatic plants and spices to season food (dried herbs such as oregano, rosemary, thyme, etc.), besides seeds (cumin, sesame, etc.), olives, and nuts, all of them being rich in a wide variety of polyphenols. In the extensive group of phenolic compounds, there are three relevant components of the MD to mention: hydroxytyrosol (HT), which is found in EVOO, resveratrol (RSV) in red grapes, and quercetin (QUE), which is contained in onions, broccoli, apples, citrus fruits and other fruits and vegetables.

HT is a phenolic phytochemical with antioxidant and anti-inflammatory properties that have been proven in clinical trials, having a positive impact on CVD prevention [224]. It has been observed that a higher concentration of HT contributes to a significant decrease in oxidized LDL and triglycerides, with a minor expression of oxidative stress-related genes as well [225]. HT is still being studied as a nutraceutical in high-fat diet (HFD)-induced obese mice models, used to visualize the reversion of inflammatory parameters (elevated TNF-α, IL-1β, IL-6) by this specific EVOO component, and also the inhibiting of the activation of TLR-4 and NK-kB pathways typical to intestinal permeability in obesity [226]. Likewise, the phenolic compounds found in EVOO, such as HT, promote the growth of Bifidobacteria, which are in part responsible for the anti-inflammatory properties in the gut [227]

RSV is a potent antioxidant and anti-inflammatory, and one of the more favored nutraceuticals these days. This nutraceutical in the gut is partly transformed by bacteria, and its derivatives are conjugated in the liver, as well as in glucuronidation and sulfation processes [228]. RSV, microbial metabolites and conjugated products can target different oxidative stress-related factors, inhibiting NF-κB and activating nuclear erythroid 2-related factor 2 (NRF2) [229], which are particularly implicated in aging [230]. RSV targets several inflammatory, metabolic and epigenetic components, and a wide range of the antioxidant enzymes implicated in gluconeogenesis, lipid metabolism, mitochondrial biogenesis, angiogenesis and apoptosis. It may block TLR4, silence pro-inflammatory genes, reduce Th17 cells and IL-17, and inhibit eicosanoid production [231]. The effects of RSV on gut microbiota have been more extensively studied in mice models. In HFD-induced obese mice, the supply of RSV denotes a decrease in the Firmicutes:Bacteroidetes ratio, avoiding *Enterococcus faecalis* expansion and allowing Lactobacillus and Bifidobacterium proliferation [232]. 

QUE, as another emblematic agent from bioactive compounds, also has multiple operational effects, including anti-carcinogenic, anti-inflammatory, antiviral, and anti-platelet-aggregation [233]. Animal models have helped us to describe the immune signaling pathways in which QUE interferes, including the inhibition of LPS, NO, PGE_2_, iNOS, COX-2, TNF-α, IL-1β and IL-6 [234], and impeding Th1 differentiation in the autoimmune disease model, but generally avoiding the accumulation of inflammatory and anti-inflammatory cells [235]. Translational research highlights its potential for use against allergies, since it may modulate Th1/Th2 balance and limit antibodies formation, and inhibit IL-8 production and histamine liberation [236]. As regards microbiota, the effects are still being studied in animal models. In colitis-affected feces from mice, the effects of QUE supplementation were observed: microbial diversity increased, the expansion of Bacteroides was promoted, and Bifidobacterium, Lactobacillus and Clostridia, acting against the reduction of Enterococcus, we promoted too [237]. The combination of diverse phytochemicals can boost their antioxidant and anti-inflammatory properties, as has been reported in some rodent models. The supply of a mix of QUE and RSV has been observed to increase microbial diversity, ameliorating dysbiosis as well as mitigating serum inflammatory markers [238]. 

### 6.3. Dietary Fiber

Dietary fiber consists of a complex of carbohydrates that are not digestible by our gut cells, but which have prebiotic effects, meaning bacteria do ferment them, giving SCFAs as the fermentation metabolites. These food components resist digestion in the small intestine, and engage in in microflora fermentation when arriving at the colon. 

In particular, the microbiota-accessible carbohydrates (MAC) contained in dietary fiber are the dietary components used by gut microbiota [239]. Indeed, MACs play a key role in the moulding of the gut microbial ecosystem; in contrast, low-MAC diets, such as WD, entail a loss of diversity [240]. Refinement in the food industry is a mistake when quality nutrients are removed, including the loss of fiber, polyphenols, and even micronutrients [241], as opposed to an MD, in which whole-grain cereals, vegetables and fruits have plenty of prebiotics [192]. Higher intakes of fiber correlate with lower arterial blood pressure and the attenuation of cardiovascular risk factors [242], a lower risk of T2DM and some cancer types (especially colorectal, gastric or esophageal), and also mortality above all [243]. Primarily, they stimulate the growth of Bifidobacteria and Lactobacilli, lifting their metabolism, and hence strengthen the gut barrier, contributing to GALT homeostasis [244].

Nevertheless, there are different types of dietary fiber, with correspondingly different physiological effects: insoluble (nonviscous) fiber, predominantly in cereal, and soluble (viscous) fiber, in fruit. The first one involves a greater addition of water to stool, with a laxative effect that the latter (which is more easily fermentable) does not offer; however, it does permit a better cholesterol lowering effect [245], but does not seem to reduce T2DM risk as much as the insoluble alternative [246]. Cereal fiber’s benefits reside in improved colonic fermentation, aiding glucose tolerance and reducing inflammation [247]. Both kinds are necessary in a balanced diet, and recommendations should be oriented towards the patients’ particular health status requirements. Another fact is the interference in energy balance and insulin homeostasis, as carbohydrates rich in fiber are low-glycemic [248].

Several controlled trials have proven that, even in the short term, the supply of fiber to individuals that have consumed high-fat and high-carbohydrate diets was able to reduce inflammatory parameters, avoiding the increase in HFD-induced LPS [249]. 

β-Glucans are the principal soluble fiber, and are the polysaccharides abundant in oat grain and in barley or wheat. Most evidence suggests that they increase the satiety sensation, and thus seems to contribute to adequate body weight control [250]. In vitro and in vivo models describe their interactions with TLR4, inducing DC maturation and attachments with other receptors on macrophages, in both inducing cytokines production, and triggering the activation of T and B cells [251]. The enhanced immunomodulatory operations start with major SCFA production, with effects on immune cells [252]. Moreover, they show antioxidant properties by reducing oxidative stress [253]. On the other hand, other common soluble prebiotics are oligosaccharides, including inulin, oligofructose (OF), lactulose, fructooligosaccharides (FOS), galactooligosaccharides (GOS), dextrin, etc., with increasing evidence of their anti-inflammatory potential. Some recent trials have showed that inulin supplementation lessens LPS and TNF-α in T2DM [254], and limits presentation of metabolic syndrome (MetS) [255]. In contrast, some studies showed that only FOS and GOS supplementation may decrease certain butyrate-producing bacteria, such as *Phascolarctobacterium* sp. or *Ruminococcus* sp. [256]. Therefore, targeting the gut microbiome for nutritional intervention, the question involves finding a balance of those fiber components in order to reach the desired beneficial effects individually.

Some non-digestible carbohydrates, such as cellulose, increase gut transition, reducing the time of colonic fermentation, and contribute to sustaining a wider microbial variety [257]. Furthermore, polysaccharides have effects on glycometabolism-related diseases [258], interacting with bacteria and the immune cells too [259], and the SCFAs derived from them strengthen gut barrier function, promoting the growth of other beneficial bacteria as well [260].

### 6.4. Vitamins 

Studies that focus on malnutrition and gut health have suggested that interactions between microbiota and the proportion of dietary vitamins are significant for immune function [167]. Vitamins are essential components that must be taken from the diet, and are crucial in microbiome dynamics [261]. These substances have multiple targets in endothelial and immune system cells.

Vitamin A (Vit A) and vitamin D (Vit D) play fundamental roles in the effective functioning of the immune system and intestinal homeostasis, modulating microbiota and strengthening barrier function [262]. Both vitamins attach to host receptors and regulate the expression of tight junctions on the intestinal epithelium. While suppressing IFNγ and IL-17 signaling from T CD4+ and inducing Treg activity, they contribute to the maintenance of microbial communities [263]. The outcome of a deficiency in these two micronutrients includes Proteobacteria increase and drastic Bacteroidetes reduction, besides the much lower expression of claudins and occludins [264].

Vit A, which is lipid soluble, seems to be the most described vitamin in terms of its pleiotropic immunomodulation effects [265]. It may be found in animal sources, such as beef liver or cheese, and in plant-based foods as provitamin A, in carrots, peppers, pumpkin or spinach, among others. Preclinical insights demonstrate that Vit A deficiency is related to disrupted BA metabolism and the advancement of *Bacteroides vulgatus* [266]. Moreover, there is a bidirectional effect between gut and microbiota that results in IgA production. SCFA acetate attaches to the GPR43 receptors on DCs, and thus these cells convert Vit A into retinoic acid (RA) [267]. This could be enhanced by a higher intake of dietary fiber, as this provides a greater amount of SCFA, and furthermore, these metabolites stimulate the activity of the retinal dehydrogenase enzyme in DC [268]. Together with Vit A intake, evidence shows that SCFA aids in preventing food allergies, reducing inflammatory responses to food antigens [269]. Therefore, SCFAs promote RA production by DC, creating an anti-inflammatory environment in the presence of IL-10 and TGF-β, increasing Treg over Th17. For its part, RA increases the conversion of Treg by inhibiting the secretion of pro-inflammatory cytokines [270,271].

Vit D, which is lipid-soluble, is a promising modulator, associated with a better response to infections, and it is used for autoimmune disease treatment [272]. It is found in few foods, so its deficiency is quite common; it is highly present in fatty fish such as salmon, tuna, mackerel, and fish liver oils, and lower concentrations of the D3 form are found in cheese and egg yolk. Animal models describe that the deficiency of it active form, 1,25(OH)2D3 (D3), reduces defences against infection, and triggers inflammatory mediators response TNF-α, IL-1β, IL-6, TGF-β and IL-17A [273]. The principal mechanism occurs when Vit D meets its receptor (VDR), which is located in B and T cells, lymphocytes, monocytes, macrophages and DC. The interaction between VDR and Vit D directly influences gut microbiota composition. In addition, bacteria metabolites may regulate Vit D and VDR at multiple levels, and orchestrate immune responses, promoting DC activity and Treg maturation, while reducing pro-inflammatory cytokines release [274]. In the same manner, VDR is highly expressed in ileon, and its signaling by Vit D is key to optimal Paneth defensins release. Thus, Vit D plays a prominent role in immune tolerance. HFD together with Vit D deficiency leads to specific defensins production and the inhibition of MUC2, causing lower tight junctions expression and thus higher gut permeability, dysbiosis, endotoxemia, systemic inflammation, fatty liver and insulin resistance [275], and typical disruptions in NAFLD, T2DM, obesity or MetS [276,277].

There are other vitamins with antioxidant potential, including vitamin C (Vit C), or ascorbic acid, and Vitamin E (Vit E), or α-tocopherol. Vit C, which is hydrosoluble (found in citrus fruits, tomatoes, red pepper and brussels sprouts), is a dietary helper for infections, as it is well leveraged by phagocytes and T cells [278]. Its best trait is its being an electron donator, behaving as an enzymes regulator. When it is found stored in phagocytes, Vit C activates the enzymes implied in phagocytosis, and enhances microbial killing [279]. Rodent models show how deficiencies in Vit C correlate with increased levels of IL-6; however, this inflammatory response does not seem to implicate gut microbiota [280]. Vit E, which is lipid-soluble (the best sources are nuts, seeds and vegetable oils), gives protection to PUFA integrity in cell membranes [281], protects intestinal mucosa against damage from ROS [282], and avoids the upregulation of cell adhesion molecules (CAMs) such as intercellular adhesion molecule 1 (ICAM1) and vascular cell-adhesion molecule 1 (VCAM1) [283,284]. Although Vit E deficiency is less common, recommendations in terms of supplementation and diet may be aimed at the elderly to boost immune competence [285], thus inhibiting the secretion of pro-inflammatory cytokines, IFNγ, IL-6 and TNF-α. Knowledge related to the gut microbiota is less supported by evidenced; recent studies in animal models suggest that low Vit E consumption alters gut microbiota composition through increasing the Firmicutes:Bacteroidetes ratio, while raising the body weight [286].

It is necessary to mention the interesting B-group vitamins, which are hydrosoluble, and which serve as a source of enzyme cofactors for the host [287]. These cannot be synthesized by mammals, so they ought to be taken from the diet or microbiota, as not all gut bacteria are able to produce them and yet they need them, as does the host [288], that is, there are auxotrophic and prototrophic bacteria. Such is the case with some butyrate-producing species from the Firmicutes phyla that are autotrophic and depend on diet and prototrophic bacteria [289]. Foods that are rich in B-group vitamins include milk, cheese, eggs, liver, meat, tuna and salmon, among others. Scarcity in any of these is related to CVD and cognitive dysfunction in aging [290]; therefore, proposals of their supplementation in the elderly are increasing, especially with folate (vitamin B9), cobalamin (B12), pyridoxine (B6) and riboflavin (B2) [291]. A broad spectrum of properties could be mentioned, and some of these will be illustrated with examples.

Thiamine (B1) (found mainly in whole-grain cereal, fish, red meat, poultry, milk and dairy products) is anti-inflammatory, activates apoptotic proteins and sparks cytochrome C liberation. Its deficiency is called beriberi, implicating T cell infiltration and inflammatory status activation with IL-1, TNF and IL-6 release [292].

Riboflavin (B2) (highly present in beef, oats, yogurt, milk and almonds) exhibits antioxidant, anti-aging, anti-inflammatory and anti-cancer features [293]. Some studies show the advantages of its supplementation in terms of disease status, such as for Crohn’s, entailing a decrease in Enterobacteriaceae [294] (Julius Z H von Martels et al. 2020), thus ameliorating dysbiosis status.

Niacin (B3) (from beef, poultry, salmon, tuna, pork, rice, peanuts, potato, etc.) has effects on lipid modification [295] that are associated with CVD risk reduction. In addition, in inflammation associated with T2DM and MetS, in which patients have increased adhesion molecules expression, a supply of B3 may help avoid monocytes’ adhesion to endothelial cells [296]. Niacin and its derivatives play a role in pro-inflammatory macrophages or M1 maturation, and contribute to polarization towards the anti-inflammatory phenotype M2 [297], a well-valued aspect of neurodegenerative disease treatment research [298] that is associated with gut dysbiosis [299].

Pantothenic acid (B5) (from beef liver, cereals, sunflower seeds, chicken, tuna, avocado, etc.) is able to regulate intestinal immunity, interfering as well with the mRNA of tight junctions, NF-kB and NRF2. An optimal supply provides the strengthening of barrier functions, increasing the expression of claudins and occludins [300]. Research suggests it is an adjuvant of host defines, and that most *Bifidobacterium* spp. and some *Lactobacillus* spp. auxotrophic for B5 take it as fuel [288]. A lack of vitamin D can lead also to a lack of pantothenic acid [301].

Pyridoxine (B6) (found in chickpeas, beef liver, tuna, salmon, chicken breast, potatoes, banana, etc.) is a cofactor for several reactions, including inflammatory signaling, such as in the kynurenine pathway [302]. This B6 has greater requirements with aging [291], and its reduced concentration contributes to depletions in T lymphocytes proliferation and differentiation [303]. More recent evidence describes B6’s interactions with mitochondrial integrity and inflammasomes [304].

Biotin (B7/B8/H) (highly present in beef liver, cooked egg, salmon and cooked pork chop), is a vitamin that consists of a ligand for several carboxylases involved in gluconeogenesis, fatty acid synthesis and amino acids metabolism. In the event of metabolic function impairment, biotin may not bind to carboxylases, and thus induces inflammation [305]. In the LPS-induced inflammation situation, a deficiency of biotin implies an enhanced secretion of proinflammatory cytokines TNF-α, IL-23, IL-1β, IFN-γ and IL-17 [306], and the greater differentiation of CD4+ T into Th1 and Th17, increasing the inflammation status [307].

Folate (B9) (rich in beef liver, spinach, black-eyed peas, rice, asparagus, lettuce, avocado, etc.) is a powerful methyl group donator, is involved in multiple processes [308], and is immunologically determinant of Treg survival, as these express folate receptor 4 (FR4). Lessening B9 induces Treg apoptosis, provoking higher intestinal inflammation [309]. Interestingly, some animal models have shown an increased Firmicutes:Bacteroidetes ratio associated with folate deficiency [310]. Recent studies have proposed the use of probiotic bacteria to increase folate production, therefore addressing gut dysbiosis associated with folate deficiencies [311]. 

Cobalamin (B12) is a potent antioxidant (predominantly in meat, fish, milk and eggs). It interferes in oxidative stress scavenging ROS [312]. Surprisingly, differences in gut microbiota have not been found between B12-deficient and non-deficient children [313]; however, it is known that bacteria do not deliver enough B12 to humans, and they compete for its derivatives [314]. Sometimes, the inconvenience of supplementation leads to the proliferation of pathogens that take advantage of B12, which reduces the expression of genes from prototrophic bacteria that synthesize the vitamin [315].

### 6.5. Trace Elements

Other micronutrients are highly present in MD, as are minerals that present multiple benefits for health, gut microbiota and the immune system. For instance, they are relevant in the protection against oxidative damage [316]. The transition metals zinc (Zn) and iron (Fe) act as cofactors furnishing the redox properties in order to facilitate T cell activation [317]. 

Zn represents almost 10% of the human proteome [318], and may be the trace element with the most robust evidence for immunomodulation [319]. Foods with high contents of this mineral may include cooked oysters, beef, crab, lobster, pork, baked beans, chicken or pumpkin. The Zn signaling pathway leads to the control of immune functions, as it targets a broad spectrum of molecules in the cells triggering proliferation, differentiation, survival and migration [320]. Its deficiencies denote a decreased number of cells of innate and adaptative immunity [321]. In mouse models, feeding with excesses of Zn could cause limitations of inflammatory events in the intestine, although no alteration in fecal microbiota [322]. Studies in humans that concern the supplementation of adjuvants for infection treatment, such as Campylobacter jejuni, are still limited [323]. Further research about microbiota–Zn interplay is still needed.

On the other hand, Fe aids the growth of commensal, but also pathogenic, bacteria in the intestine, possibly increasing inflammation. Furthermore, under inflammation conditions, as in IBD, iron metabolism is disrupted, causing anemia in the host [324,325]. Moreover, Fe deficiency spoils Th1 activity [326]. The side effects of not absorbing the excess of Fe in the intestinal lumen will have consequences for host–healthy microbes interplay [327]. Recent studies have focused on the possibility of reducing the iron availability to gut microbiota in the colon, with the aim of reducing pathogen growth [328]. Furthermore, the Bifidobacteriaceae family has the ability to bind iron in the large intestine, thus attenuating the damage caused by the free radicals produced in iron metabolism [329]. For this reason, and as discussed in the case of polyphenols above, vitamin or mineral deficiencies may be benefited from supplementation, not only with these substances but also with probiotics.

Non-metal selenium (Se), which also has redox features, displays immunobiological activity when binding to selenoproteins [330], and has special potential in its resistance to viral infections [326]. Selenoproteins are key in ROS modulation, and influence lymphocyte activation, proliferation and differentiation [331], but not all types of immune responses are well described yet [332]. Some studies have demonstrated that microbial diversity increases in the presence of dietary Se [333]. Further research is still needed to comprehend selenoproteins–microflora interactions.

To sum up, MD counts on a wide variety of nutritional components that are clearly beneficial for the proper functioning of the gut microbiota and immune system, as represented in Table 1. These effects are prominently contrary to those reported by WD, as will be discussed below.

## 7. Western Dietary Pattern as a Model of Unhealthy Eating

Contrary to the MD, WD represents a global concern, and is responsible for the obesity pandemic and NCDs, including cancer, CVDs, osteoporosis, autoimmune diseases or T2DM, among others [334]. WD are characterized by a high content of unhealthy fats, refined grains, sugar, salt, alcohol and other harmful elements, along with a reduced consumption of fruits and vegetables. This leads to critical changes in both gut microbiota and immune system, negatively affecting the gut integrity, and thus promoting local and systemic chronic inflammation [335,336]. 

To understand the unfavorable effects of WDs, it is crucial to know the concept “food matrix”, which, in a simple manner, states that the different compounds located in the food interact in a coordinated way in the human body, determining the positive or negative effect of an aliment [337]. Thus, although they have similar contents of macronutrients, MD represents a protective factor for obesity and other NCDs, while WD is clearly a risk factor [338]. Ultra-processed food and drinks (UPFDs), designated by the NOVA classification based on the nature, extent and purpose of processing [339], are considered a major hallmark of WDs, and a higher consumption of these components appears to be related with an increased risk of morbidities [340,341,342] and mortality [343], although further studies are needed to establish causality. UPFDs are highly profitable, hyper-palatable and ready-to consume products, mainly composed of the non-habitual ingredients of “real food” (e.g., hydrogenated/de-esterified oils or additives designed to provide the previous characteristics mentioned) [344]. In addition, the other deleterious elements found in the WD pattern, such as added sugars, carbohydrates and saturated fat, are abundantly found in UPFDs, while beneficial micronutrients, such as previously reported vitamins A, C, D, E and trace-elements such as zinc, phosphorus, calcium, magnesium or potassium are inversely related with their consumption [345]. UPFDs are equally a worrisome issue for children, who are a vulnerable population, not only as regards their consumption, but also in terms of suffering their negative effects [346,347]. The poor matrix of these foods combined with their reduced fiber contents generates an unfavorable environment in the gut and the microbiome, therefore leading to dysbiosis and immune alterations [348,349]. Thus, proper education, awareness and measures are needed from a public health perspective. Here, we will summarize the evidence regarding the different elements of WD and UPFDs so as to shed light on their relationship with gut microbiota and immune system status. 

### 7.1. Refined Carbohydrates

When analyzing unhealthy elements of WDs and UPFDs, refined carbohydrates, particularly added sugar and processed grains (such as white flour or white bread) are elements of note in the negative modulation of the gut microbiota and immune system. Added sugars are all those sugars that are present in foods, mainly UPFDs, or are naturally present in unsweetened fruit juices, honey and syrups [350]. Added sugars are considered empty calories, as they may substitute the intake of components with higher nutritional interest, and their consumption is prominently associated with negative cognitive functioning and addiction [351,352]. Fructose overconsumption, prominently through excessive refined sugars intake, is associated with a systemic pro-inflammatory status, and is also related with cortisol hyperactivation, increased visceral adiposity, and insulin resistance [353]. In addition, the deleterious effects of the consumption of added sugars, and particularly from sugar-sweetened drinks, have been reported in the gut microbiota, promoting an increased Firmicutes/Bacteroidetes ratio and reducing the proportion of favorable butyrate-producers such as *Lachnobacterium* [354]. Likewise, added sugars provoke augmented gut permeability and endotoxemia, thereby leading to inflammation and systemic complications [355]. Finally, there are inconsistent data when analyzing the consumption of refined versus whole-grain food. Thus, some studies have reported the favorable results obtained from whole-grains consumption in both gut microbiota and the immune system [356], although other research has found a positive effect on inflammation in a gut microbiota-independent manner [357]. Nonetheless, further research is needed to better understand the role of refined grains in human health and pathology [358]. 

### 7.2. Unhealthy Fats

High-fat diets (HFD) typical of western dietary patterns are equally one of the main concerns of unhealthy eating. In a murine model of C57BL/6 mice fed with a high-fat diet (HFD), the microbial composition was different to that of other mice fed with low-fat diets (LFD). HFD mice showed quite elevated Firmicutes:Bacteroidetes ratios, minor antimicrobial Paneth activity, and higher pro-inflammatory IFNγ, TNFα, IL-1β and IL-6 cytokines concentrations [359], along with bacterial translocation leading to endotoxemia [360]

Contrary to classical beliefs, saturated fats are not the main enemy of a healthy diet. In fact, they could be included as part of one [361]. On the other hand, trans fats are mostly considered a negative or harmful fat, as they are frequently present in UPFDs [362], although they could be found naturally in ruminants-derived products, acting as a precursor of linoleic acid, a beneficial lipid [363], showing again that the method of processing and the food matrix, rather than the nutrients, provide the benefits or dangers derived from food consumption. Trans fatty acids intake in the context of WD has been reported as a potential cause of gut dysbiosis [364]. As such, unhealthy fats have been related to gut dysfunction, including the disruption of enteroendocrine cells [365] and the promotion of intestinal permeability and inflammation, mainly affecting critical gut microbiota populations and LPS [182]. The established endotoxemia could be directly associated with low-grade chronic inflammation, thus conducting to the development of NCDs [366]. Microbial regulators of BA are also potentially affected by unhealthy fats, which may aid in the establishment of certain conditions, such as obesity [367].

WD and UPFDs are both frequently rich in refined oils, which may have profound impacts not only from a health perspective, but also in terms of sustainability [368]. A classic example is palm oil (PO), the most exploited oil worldwide, which represents a threat to ecological sustainability due to the combination of UPFD and unhealthy products contributing to a higher risk of NCDs [369]. Contrary to non-refined PO, which is composed of saturated fats (mainly palmitic acid) but also contains many antioxidants (essentially tocotrienols and tocophenols), refined PO presents quite a reduced content of these antioxidants, and therefore the quality of the oil is reduced too [370]. In this sense, refined PO seems to have powerful negative effects on the gut microbiota, decreasing the abundance of *Akkermansia muciniphila*, SFB, and *Clostridium leptum*, leading to the release of pro-inflammatory cytokines and the loss of intestinal integrity [371]. Refined olive oil, contrary to non-refined EVOO, is associated with a growth of non-beneficial bacteria from the *Desulfovibrionaceae*, *Spiroplasmataceae*, and *Helicobacteraceae* families, along with a reduction in favorable *Erysipelotrichaceae* and *Sutterellaceae*, with negative implications for the host’s immune system [372]. In the same line, Rodríguez-García et al. [373] showed in animal models that sunflower oil induced Sphingomonas and *Neisseria* spp., while limiting the growth of *Akkermansia muciniphilla* and Bifidobacterium. In the same way, coconut oil restricts *Akkermansia muciniphilla* abundance, while enhancing *Staphylococcus, Prevotella* and *Bacteroides* spp. Interestingly, both microbial changes were associated with a pro-inflammatory status, increasing the risk of colorectal cancer. 

Furthermore, increased omega-6 fatty acid levels are typically associated with WDs, particularly due to refined oils and UPFD, increasing the omega 6:omega 3 ratio up to 15:1 [374] and enhancing metabolic endotoxemia through direct interactions with gut microbiota [375]. In this sense, the altered omega 6:omega 3 ratio has been found to promote the development of Enterobacteriaceae, segmented filamentous bacteria and *Clostridia* spp., leading to a pro-inflammatory environment that could be attenuated by the introduction of omega 3 PUFA [376]. In the same manner, WD is associated with a reduced production of PUFA metabolites, probably through the detrimental effects of this diet on the microbiota [212].

### 7.3. Excessive Meat Consumption and Fast Food

Red meat, and prominently processed meats, are similarly associated with WD patterns. There are many mechanisms that have been proposed in the relationship between processed and red meats and certain pathological conditions, especially as concerns colorectal cancer (CRC) risk, some of which involve the gut microbiota [377]. Among them, red and processed meats contain high levels of L-carnitine, which, along with choline, betaine and lecithin, are considered the precursors of a critical product known as trimethylamine (TMA) by the gut microbiota [378]. Then, TMA is transported to the liver, where it is transformed to trimethylamine N-oxide (TMAO), which is associated with inflammatory pathways and CVD risk [379]. Although TMAO itself might not be considered a negative metabolite, a higher amount of this component is associated with unfavourable outcomes in a context of unhealthy dietary patterns, as TMA is mostly synthesized by Firmicutes and Proteobacteria, two phyla increased with gut dysbiosis [380]. On the other hand, betaine is prominently found in some healthy plant-based components such as spinach and beets, and choline may be found in positive animal-based products like eggs, milk and fish [381]. Interestingly, fish and seafood may also contain TMA and TMAO, having been proved to have conversely anti-inflammatory properties in some studies [382]. These findings show that gut dysbiosis promoted by diet may collaborate with higher levels of TMA and TMAO, leading to pro-inflammatory conditions, but when TMA is not produced by an altered gut microbiota, it may present anti-inflammatory properties, thus demonstrating the direct influence of gut microbiota over immune system status.

Nonetheless, red and processed meats contain elevated levels of negative components, such as heme iron, which has been correlated with an hyperproliferation of colonic enterocytes and the alteration of the intestinal barrier [383]. In particular, changes in *Fusobacterium nucleatum*, *Streptococcus bovis/gallolyticus*, *Escherichia coli*, and *Bacteroides fragilis* have been reported due to excessive red meat consumption, interacting with the other negative factors contained in red meats, including proper heme, N-nitroso compounds and heterocyclic amines [384]. Another key element contained in red and processed meats, N-glycolylneuraminic acid (Neu5Gc), could also be regulated by specific gut microbiota bacteria, thereby increasing or reducing the pro-inflammatory effects of this substance, known as xenosialitis [385]. 

Fast food is another key element in the western dietary pattern, and it should be addressed not only from a biological perspective, but also from socioeconomic and psychological approaches [386]. When comparing Mediterranean vs. fast food diets, even over a short time (4 days), critical changes in the gut microbiota have been noted. In particular, there was an augmentation in BA-tolerant bacteria, along with a decrease in SCFA and indole derivate producers [387]. The excessive BA caused by western dietary patterns may be associated with a disruption in the intestinal barrier, therefore promoting a pro-inflammatory environment [388], and under inflammatory and dysbiosis conditions, tryptophan is transformed, via the enzyme indoleamine 2,3-dioxygenase-1 (IDO-1), to kynurenine by the host cells. As such, the reduced levels of tryptophan are related to a reduction in the levels of IL-22, leading to increased intestinal permeability and LPS translocation [389]. This is added to the reduced production of SCFAs, which together show the negative role of fast food and WDs in the gut microbiota and immune system. 

### 7.4. Salt and Additives. 

Although the recommended values of salt are established at 5 g/day, the available evidence has demonstrated that a reduced intake of salt should be recommended in the context of health [390]. In this context, different studies have implicated UPFD in the overconsumption of salt, contributing to the appearance of hypertension and CVD [391]. In this sense, Wilck et al. [392] have proven the direct effects of high salt levels in the gut microbiota-Th17 axis, particularly in reducing the populations of *Lactobacillus* sp., which could be associated with the altered production of SCFA identified in high-salt diets [393,394]. Other populations negatively affected include *Oscillibacter*, *Pseudoflavonifractor*, *Clostridium* XIVa *Johnsonella* and *Rothia*, while other species are increased, including *Parasutterella* spp. *Erwinia genus*, *Christensenellaceae*, *Corynebacteriaceae Lachnospiraceae* and *Ruminococcus* [395], which could aid in explaining the unfavorable effects of high-salt diets on the organism

Additives are another crucial element of western dietary patterns and UPFD. Despite the fact that some additives could be considered innocuous, an increased amount of studies have identified the negative effects of food additives on gut homeostasis [396]. The most studied food additives are artificial sweeteners and sugar alcohols. Low/non-caloric sweeteners, such as sucralose, are associated even at low doses with gut permeability, inflammation and gut microbiota changes, promoting reductions in beneficial bacteria (Lactobacillus and Bifidobacteria) along with an increase in pathogenic bacteria (Enterobacteria) [397]. Further research is needed to understand how other artificial sweeteners interact with the gut microbiota and immune system, although observational studies seem to indicate similar negative results, particularly for acesulfame K and aspartame [398,399,400]. Sugar alcohols (e.g., maltitol, xylitol, sorbitol, erythritol) appear to act favorably on gut microbiota composition, acting similarly to prebiotic compounds. The roles of the rest of the additives in terms of gut microbiota and immune system remain to be fully elucidated [401]. 

Here, we show the main impacts of WD on gut microbiota and the immune system, as summarized in Table 2. Overall, these nutritional components are doubly hazardous: On the one hand, they lead to unfavorable changes in the organism, replacing healthy nutrients such as those contained in MD. On the other hand, these nutrients are contained in a poorer food matrix, with synergic harmful effects explaining the negative interplay between WD, gut microbiota and immune system. 

## 8. Conclusions and Future Directions

**Figure 3:**
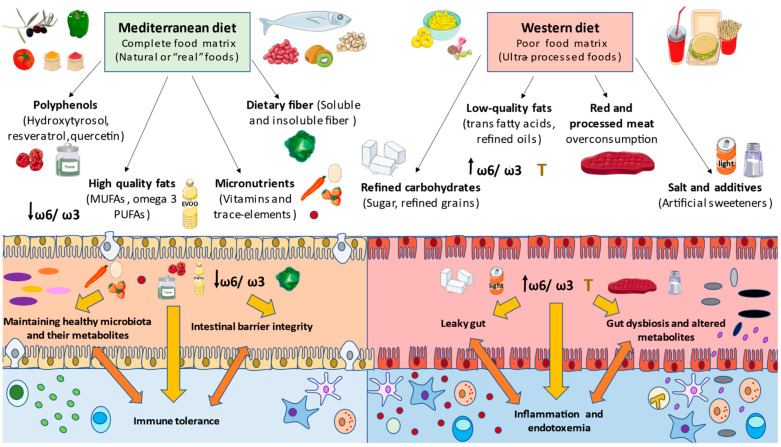
A general overview of the main nutritional components modulating both gut microbiota and immune system. As it is represented, the Mediterranean diet, rich in plant-based aliments, presents polyphenols, high-quality fats (MUFAs and high omega 3 PUFAs), micronutrients, such as vitamins and trace elements, and dietary fiber that, in an adequate and complete food matrix, will determine their beneficial properties in maintaining gut microbiota eubiosis and its metabolic products, along with intestinal barrier integrity and immune tolerance. On the other side, western diets and ultra-processed foods, characterized by low levels of dietary fiber or micronutrients, present a plethora of nutritional components, including refined carbohydrates (sugar and refined grains), low-quality fats (trans fatty acids and an excessive omega 6/omega 3 ratio due to the refined oils), salt and unhealthy additives (mainly sweeteners), and finally excessive red and processed meat consumption. Moreover, they comprise a poor food matrix that will have detrimental effects at the intestinal barrier, leading to leaky gut, gut dysbiosis and altered metabolites, further leading to a local inflammation and the presence of LPS in the bloodstream that will contribute to systemic endotoxemia and chronic inflammation.

The gut microbiota is considered by some authors as a single organ [402], playing critical roles in host homeostasis, and different microbial communities may directly influence the immune system. As summarized in Figure 3, diet must be the most important environmental factor positively or negatively affecting both gut microbiota and immune system, although many other elements should be considered to fully understand this complex interplay. 

As described, each dietary component has a direct impact on host health through the intestinal epithelial barrier, commensal bacteria and thus cell immunophenotypes, and their pro-inflammatory or anti-inflammatory responses. Due to their beneficial properties, some nutritional components found in nutrient-rich and high-quality balanced diets like MD keep offering valuable information in terms of the clinical management of the NCDs burden. With the aim of leveraging the anti-inflammatory and antioxidant potential of polyphenols, nutraceuticals research based on these phytochemicals seems to be of great interest [403], thus improving their bioavailability and absorption properties, and even facilitating glucuronidation or sulfation forms as well, these being biologically active for patients with metabolic disorders. Further work is warranted in immunotherapy with vitamins as well, as these are promising adjuvants for a wide spectrum of diseases, including inflammatory diseases [404], cancer [405] and depression [406], as well as in the prevention of these NCDs, among others.

The human being is more susceptible to infections in the first or last years of life, and even more so without a proper diet [407]. The period of the first stage of life seems to be critical in the maturation of the immune system, and is also named the “window of opportunity” [6], having an impact not only on physical development but also on neurocognitive and disease risks [408]. On the one hand, in the early life, nutritional strategies are pursued in order to prevent NCDs such as asthma or allergies in adulthood [409], which involve considering breastfeeding [410], or including prebiotics or human milk oligosaccharides (HMO) in formula feeding for infants [411,412], as well as providing fiber and PUFA to the mother during pregnancy [413]. All these nutrients will favor the epigenetic mechanisms that may have an effect in later life, and which will shape the immunophenotype differentiation to Th1, Th2, Th17 or Treg [414]. Despite the fact that the food industry makes advances in infant formula products, as a result of evolution, breastfeeding is the first contact of the human body with natural prebiotics. Likewise, other components from human milk interact with the baby’s immune system, for example PUFAs, cytokines, allergens, immunoglobulins, and chemokines [415], although some Bifidobacterium species are also able to take glycans from the mother’s milk [416]. 

On the other hand, aging is another critical event in which gut microbiota may have a central role, as immune competence shrinks with aging, an event referred to as immunosenescence and inflammaging [417]. Focusing on healthy aging, the current studies are attempting to ameliorate quality of life in the elderly [418]. In this line, neurodegeneration and the associated neuroinflammation cause reductions in the diversity of beneficial bacteria, such as Lactobacilli [419,420], and debunk the hypothesis that microbes have a direct impact on host aging [421]. 

In summary, the diet modulates both immune system and gut microbiota at the same time, establishing a bidirectional dialogue with signaling pathways or metabolite production, which affect each other functions.

In the event of malnutrition, deficiencies in micronutrients are quite common, and determinant of the physiopathology of immunodeficiencies and inflammatory diseases. Such are the cases of NCDs, obesity, T2DM, MetS, CVD, and IBD, among others. These maladies are typically prevalent in developed countries, where WD patterns are the norm. WD is characterized by high carbohydrate and saturated fat intakes, and includes a low-quality “food matrix”, which is refined and ultra-processed with added sugars and additives. In contrast, adherence to an MD may ameliorate inflammation and gut microbiota dysbiosis, thanks to its abundance in PUFAs, dietary fiber, polyphenols, vitamins and trace elements, all necessary for achieving an adequate balance of Th17/Treg in the host and guaranteeing high microbial diversity.

Nutritional deficiencies should be addressed personally, evaluating the patient’s health status and making some recommendations not only as regards supplementations, but also in terms of healthy dietary habits. However, dietary components cannot be treated individually. Deficiencies in any micronutrient, and sometimes also excesses, can be crucial for the outbreak of an opportunistic pathogen and the reduction of beneficial bacteria, such as Lactobacillus and Bifidobacterium. Therefore, in malnutrition treatment, targeting microbiota reprogramming involves probiotics together with the nutritional component that is scarce, as certain bacteria help those nutraceuticals to grow, and provide SCFA or other metabolites to achieve host immunocompetence.

## Figures and Tables

**Table 1:** The role of dietary components abundant in MD in the gut microbiota, immune system, intestinal barrier, and systemically.

Nutrient	Top Food Source	Recommended Intakes	Immunomodulation	Gut Microbiota Modulation	Excess/ Deficit Effects	Effect on Epithelial BARRIER	Other Physiological Effects Affecting Immunocompetence	References
MUFAs: oleic acid	EVOO, olive oil, olives	______	↓ IL-6, IL-17A, TNF-α, IL-1β, COX-2	↑ Bifidobacterium and Lactobacillus ↑ Butyrate production	Excess: ↓ number of total bacteria	Keeping integrity	↓ LDL-c, LDL-c oxidized and blood pressure. Anti-inflammatory, atheroprotective, colonocytes protection against oxidative stress	[194,195,196,197,198,199,200,201,202,203]
PUFAs	Vegetable oils, nuts, fish				Elevated ω-6: ω-3 ratio (>4:1) ↑ inflammation	-	ω-6: ω-3 ratio (<4:1): ↓ inflammation MD↓ratio (2:1/1:1): ↑ anti-inflammation	[203,204] [209,210]
ω-3	Fish, seafood, nuts, seeds, plant oils, eggs, dairy products	1.6 g (male) 1.1 g (female)	CLA: ↑ Treg production ↑ NF-α, IL-1β, and IL-6 ↓ IL-10 and PPAR-γ	Balance Firmicutes:Bacteroidetes ratio ↓ LPS-producing Enterobacteria Can produce CLA and CLnA: ↑ Ruminococcus and Prevotella				[206] [211,212,213,214,215,216]
ω-6	Vegetable oils, nuts, seed, soy derivates like tofu, eggs or poultry		ARA is a precursor of pro-inflammatory molecules: eicosanoid hormones prostaglandins or leukotrienes					[207,208]
Polyphenols (flavonoids and non-flavonoids)	Berries anthocyanins, olives, nuts tannins aromatic plants and spices (dried herbs like oregano, rosemary, thyme, etc.), seeds (cumin, sesame, etc.)	______	↓Pro-inflammatory molecules	Prebiotic effect: ↑ *Lactobacillus* spp. ↓ *Enterococcus* *caccae* ↓ Dysbiosis ↑ Microbial diversity		Keeping semipermeable character: some ↑ tightjunctions and some ↓	Antioxidant and anti-inflammatory	[217,218,219,220,221,222]
HT	EVOO	______	Inhibits TLR-4 and NF-kB ↓ TNF-α, IL-1β, IL-6 in inflammation status	↑ Bifidobacterium		Keeping integrity	Anti-inflammatory, antioxidant, cardio-protection, ↓ ox-LDL, TG, oxidative stress	[224,225,226,227]
RSV	Red grapes	______	Inhibit NF-kB Block TLR4 ↓ Th17, IL-17, eicosanoids	↓ Firmicutes: Bacteroidetes ↓ *Enterococcus faecalis* ↑ Lactobacillus and Bifidobacterium		Keeping integrity	Antioxidant, anti-inflammatory ↓ oxidative stress (+) NRF2: anti-aging	[228,229,230,231,232]
QUE	Onions, broccoli, apple, citrus fruits and vegetables	______	↓ LPS, NO, PGE_2_, iNOS, COX-2, TNF-α, IL-1β, IL-6 ↓ Th1 Modulation Th1/Th2 balance	Bacteroides, Bifidobacterium, Lactobacillus, and Clostridia against reduction of Enterococcus		Keeping integrity	Anti-allergic potential, anti-inflammation	[233,234,235,236,237,238]
Dietary fiber	Fruits and vegetables	25–30 g per day	↓ Inflammatory parameters	SCFA production ↑ Microbial diversity ↑ Bifidobacteria and Lactobacilli		Strengthening	↓ Blood pressure ↓ Risk of CVD, T2DM, MetS, colorectal and gastric cancers Better energy balance	[239,240,241,242,243,244] [192] [248,249]
Soluble: β-Glucans Oligosaccharides (inulin, OF, lactulose, FOS, GOS, dextrin)	Fruit Oat grain, barley, wheat		DC maturation (+) B and T cells ↓ LPS, TNF-α	=	Only FOS and GOS supplementation ↓ Some butyrate-producing bacteria (e.g., *Ruminococcus sp*)	Strengthening	↑ Satiety sensation Antioxidant: ↓ Oxidative stress ↓ c-LDL	[245] [250,251,252,253,254,255,256]
Insoluble:	Cereal		=	= ↑ Colonic fermentation	Excess is laxative	Strengthening	Glucose tolerance Anti-inflammatory ↓↓ T2DM risk	[246,247] [257,258,259,260]
Vitamins			Pleiotropic				Pleiotropic	[187,188] [261]
Vitamin A	Animal sources like beef liver or cheese, and in plant-based foods as provitamin A, in carrots, peppers, pumpkin, spinach	900 µg (Including pro-vitamin A carotenoids)	↓ IFNγ and IL-17 ↑ Treg	Maintain diversity ↓ Firmicutes:Proteobacteria ratio	Deficiency --> Disrupted BA metabolism ↑ *Bacteroides vulgatus* and ↓ tight junctions (claudins and occludins)	Strengthening: ↑ tight junctions	Anti-inflammatory Allergy prevention	[262,263,264,265,266,267,268,269,270]
Vitamin D	Highly in fatty fish like salmon, tuna, mackerel, and fish liver oils; lower concentrations of D3 form are found in cheese and egg yolk	15 µg	↓ IFNγ and IL-17 ↑ Treg (+) VDR: Treg maturation ↓ Pro-inflammatory cytokines ↑ Tolerance	Maintain diversity: ↓Firmicutes: Proteobacteria ratio	Deficiencies: ↓ MUC2 ↓ Tight junctions (Claudins and occludins) Dysbiosis Endotoxemia Bad response against infections (↑ TNF-α, IL-1β, IL-6, TGF-β, IL-17A) Fatty liver Insulin resistance ↓ B5	Strengthening: ↑ Tight junctions Optimal Paneth defensins release		[262,263,264] [272,273,274,275,276,277]
Vitamin C	Citrus fruits, tomatoes, red pepper and brussels sprouts	90 mg (male) 75 mg (female)	Helps T and phagocytes in infections	↑ Killing of pathogens	Deficiencies ↑ IL-6	Keeping integrity	Antioxidant	[278,279,280]
Vitamin E	Nuts, seeds and vegetable oils	15 mg	↓ IFNγ, IL-6, TNF-α		Deficiency in elderly Excess ↑ Fermicutes:Bacteroidetes	Protects barrier from ROS	Antioxidant Protects PUFAs in cell membranes ↓ CAMs Anti-aging	[281,282,283,284,285]
B-group vitamins	Cheese, eggs, liver, meat, tuna, salmon				Deficiencies in any of them, especially B9, B12, B6 and B2 CVD, and cognitive dysfunction in aging		Cofactors for enzymes	[287,288,289,290,291]
Thiamin (B1)	In whole-grain cereal, fish, red meat, poultry, milk and dairy products	1.2 mg (male) 1.1 mg (female)	Immune homeostasis		Deficiency = beriberi Inflammation ↑ IL-1, TNF, IL-6		Anti-inflammatory (+) Pro-apoptotic proteins	[292]
Riboflavin (B2)	Beef, oats, yogurt, milk and almonds	1.3 mg (male) 1.1 mg (female)	↓ Inflammatory parameters	↓ Dysbiosis ↓ Enterobacteriaceae	-		Antioxidant, anti-aging, anti-inflammatory and anti-cancer	[293,294]
Niacin (B3)	Beef, poultry, salmon, tuna, pork, rice, peanuts, potato	16 mg (male) 14 mg (female) Including niacin equivalents intake	↓ M1 macrophagues ↑ M2 macrophagues	-	-	-	↓ Ox-LDL ↓CAMs ↓ CVD risk ↓ T2DM and MetS risk	[295,296,297,299]
Pantothenic acid (B5)	From beef liver, cereals, sunflower seeds, chicken, tuna, avocado	5 mg	Interacts with mRNAs of NF-kB and Nrf2	Most *Bifidobacterium* spp. and some *Lactobacillus* spp. use it as fuel		Strengthening Interacts with mRNAs of tight junctions, ↑ claudins and occludins		[288] [300,301]
Pyridoxine (B6)	Chickpeas, beef liver, tuna, salmon, chicken breast, potatoes, banana	1.3 mg	Involved in kynurenine pathway Interacts with inflammasomes	-	Deficiencies with aging: ↓ T proliferation and differentiation	-	Mitochondrial integrity	[291] [302,303,304]
Biotin (B7/B8/H)	Found in beef liver, cooked egg, salmon and cooked pork chop	30 µg			Deficiency: ↑ TNF-α, IL-23, IL-1β, IFN-γ and IL-17 ↑ Th1 and Th17		Cofactor of carboxylases involved in gluconeogenesis, fatty acid synthesis and amino acids metabolism	[305,306,307]
Folate (B9)	Beef liver, spinach, black-eyed peas, rice, asparagus, lettuce, avocado	400 µg	Treg survival		Deficiency: Treg apoptosis ↑ Intestinal inflammation Dysbiosis		Methyl groups donator so important in epigenetics	[308,309]
Cobalamin (B12)	Meat, fish, milk and eggs	2.4 µg			Excess ↑ pathogens proliferation		↓ ROS	[312,313,314,315]
Trace elements			Cofactors with redox properties that facilitate T cell activation				Protection against oxidative stress	[316,317]
Zn	Cooked oysters, beef, crab, lobster, pork, baked beans, chicken or pumpkin	11 mg (male) 8 mg (female)	Modulates NF-kB pathway, controls oxidative stress and is implicated in anti-inflammatory and pro-inflammatory responses	-	Deficiencies ↓ number of immune cells	-	Involved in multiple cell events: proliferation, differentiation, survival and migration	[318,319,320,321,322,323]
Fe	Meat, seafood, nuts and beans	8 mg (male) 18 mg (female)		Bifidobacteriaceae family helps binding iron ↓ Damage from free radicals	Abundancy in intestine, causes pathogenic bacteria to proliferate, causing anemia in host ↑ Inflammation Deficiency disrupts Th1 activity	-	-	[324,325,329]
Se	Seafood and meat	55 µg	Binds to selenoproteins and has potential in resistance to viral infections Influence lymphocyte activation, proliferation and differentiation	Seems dietary Se ↑ microbial diversity			Redox reactions: Selenoproteins play a role in ROS modulation	[326,330,331,332,333]

Recommended intakes are expressed for average adults, they may vary in childhood, pregnancy, lactation or the elderly, or depending on pathological status. In some cases, like polyphenols, there is no specific evidence for recommended dosage. MUFAs = monounsaturated fatty acids; EVOO = extra virgin olive oil; COX-2 = cyclooxygenase-2; PUFAs = polyunsaturated fatty acids; MD = Mediterranean diet; CLA = conjugated linoleic acids; CLnA = conjugated linolenic acids; ARA = arachidonic acid; HT = hydroxytyrosol; RSV = resveratrol; QUE = quercetin; PGE_2_ = prostaglandin E2; OF = oligofructose; FOS = fructo-oligosaccharides; GOS = galacto-oligosaccharides.

**Table 2:** The role of dietary components abundant in WD in the gut microbiota, immune system, intestinal barrier, and systemically.

Nutritional Component	Top Food Sources	Maximum Intake Limits Per Day/Week	Immunomodulation	Gut Microbiota Modulation	Intestinal Barrier Damage and Other Pathological Effects	Alternatives/Substitutes and Suggestions	Ref
Added/Free sugars	UPFDs or naturally present in unsweetened fruit juices, honey and syrups	<25 g (↓ 10% of total daily Caloric intake)	Pro-inflammatory environment	↑ Firmicutes/Bacteroidetes ratio, ↓ population of favorable butyrate producers (e.g., *Lachnobacterium* sp.)	Intestinal barrier disruption, endotoxemia, insulin resistance, visceral adiposity, cognitive dysfunction and addiction	Replacing added sugars consumption by intrinsic sugars (Whole fruits and vegetables) Using healthy sweeteners: Cinamon, vanilla, dates, raisins, banana, pumpkin, sweet potato, etc.	[350,351,352,353,354,355]
Refined grains	White bread, UPFDs, white flours and derivates	______________	↑ IL-6 and CRP levels; negative functioning in memory T cells	Probable effects ↑ Enterobacteriaceae while ↓ SCFA producers (Lachnospira)	Further evidence is needed to determine the possible effects of refined grains on the human body	Substituting refined with whole-grain products. Combining refined grains products consumption with fiber-rich vegetables and fruits may reduce their detrimental effects	[356,357,358]
Unhealthy fats	UPFDs, animal products, fast food	Daily fat recommendations is ≤ 30% of total Caloric intake	↑ pro-inflammatory cytokines IFNγ, TNFα, IL-1β and IL-6	↑Firmicutes/Bacteroidetes ratio	↓ Antimicrobial Paneth cells activity	Limiting the consumption of these fats by previous reported healthy fats (MUFAs, PUFAs) should be the basis of fat intake	[359,360]
Saturated fats	Animal-derived products (Milk, butter, meat, etc.) and plant-based foods (chocolate, nuts, cocoa, coconut and palm oils)	Not superior to 10% of daily intake	______________	______________	______________	Saturated fats are not as dangerous as thought before. It is more important to observe the quality and processing of the food, even if it presents a high content of saturated fats (e.g., high % dark chocholate, nuts or milk)	[361]
ω-6/ ω-3 ratio	Previously reported	ω-6/ ω-3 ratio must not exceed 4/1 proportion	Pro-inflammatory status	↑ Enterobacteriaceae, Segmented Filamentous Bacteria and Clostridia.	↑ Metabolic endotoxemia ↓ Production of PUFAs metabolites	Increase the intake of omega 3-enriched foods and reduce omega 6 foods	[212], [374,375,376]
Trans fats	Naturally in ruminant meats and UPFDs	Less than 1% of daily caloric intake	↑ Low grade chronic inflammation	Promotion of gut dysbiosis, particularly affecting BA producers bacteria	↑ Intestinal permeability, disruption of enteroendocrine cells	As it is prominently associated with ultra-processing, it should be avoided	[182], [362,363,364,365,366,367]
Refined oils	UPFDs, precooked foods	______________	Pro-inflammatory cytokines↑	Refined palm oil ↓ *Akkermansia muciniphila*, SFB, and *Clostridium leptum* growth Refined olive oil ↑ Desulfovibrionaceae, Spiroplasmataceae, and Helicobacteraceae along with Erysipelotrichaceae and Sutterellaceae ↓ Sunflower oil ↑ Sphingomonas and *Neisseria* spp. while ↓ *Akkermansia muciniphilla* and *Bifidobacterium* spp. Coconut oil ↓ *Akkermansia muciniphilla* abundance and ↑ *Staphylococcus*, *Prevotella* and *Bacteroides* sp.	↑ Intestinal permeability	Cooking with EVOO, or non-refined oils; look carefully at nutritional labeling, reduce precooked foods	[368,369,370,371,372,373]
Red and processed meats components over-consumption	Beef, veal, pork, lamb, mutton, horse, or goat meat consumed directly or after further processing (sausages, corned beef, and biltong or beef jerky)	______________	Neu5Gc contained in red meat, N-nitroso compounds TMA produced by red and processed meat precursors under conditions of dysbiosis may exert pro-inflammatory effects	Changes in certain microbial populations like *Fusobacterium nucleatum, Streptococcus bovis/gallolyticus*, *Escherichia coli*, and *Bacteroides fragilis*	Altering intestinal barrier ↑ Hyperproliferation of colonic enterocytes and correlation with colorectal cancer	Red meat is not a central component of the human diet and it could be completely displaced. However, it might be equally included in a healthy diet, although moderately (Not superior to 2 to 3 portions per week or 350–500 g according to AICR guidelines) Avoid or further limit processed meats consumption	[377,378,379,380] [383,384,385]
Salt	UPFDs	<5 g per day	Affecting gut microbiota-Th17 axis	↓ *Lactobacillus* sp. Oscillibacter, Pseudoflavonifractor, Clos-tridium XIVa Johnsonella and Rothia ↑ *Parasutterella* spp. *Erwinia genus*, Christensenellaceae, Corynebacteriaceae Lachnospiraceae and Ruminococcus	Promoting hypertension and CVD risk	Avoid consumption of high-salt foods, particularly ultra-processed. It is possible to cook with salt, but at moderate doses. Maintaining a proper balance of Na+ and K+ should help to avoid systemic defects of salt	[391,392,393,394,395]
Additives	UPFDs Processed foods	______________	Low/non caloric sweeteners are associated with a pro-inflammatory switch	↓ Lactobacillus and Bifidobacteria microorganisms; ↑ pathogenic bacteria Polyalcohols may act favorably in gut microbiota, acting like prebiotics ↑ Bifidobacterium and Lactobacillus	↑ Gut permeability, glucose intolerance, weight gain ↑ Gut integrity, no negative effects at cholesterol, glucose or insulin levels	Additives are a difficult component of diet to study, and most of them are inocuous or necessary for food conserving. However, some of them have provided accumulative evidence of their potential negative effects, so avoiding their consumption should be suitable	[396,397,398,399,400,401]

Maximum intake suggested has been extracted from World Health Organization (WHO) recommendations, although it could be favorable to even reduce these limits. Some alternatives and advice are given to avoid the overconsumption of these detrimental components, in order to progressively replace unhealthy with healthy ingredients.
